# Supplementary material for: A non-pyrophoric precursor for the low temperature deposition of metallic aluminium
Source: Nat Commun. 2025 Jul 1;16:5645. doi: 10.1038/s41467-025-60786-2 (PMC12215716; doi:10.1038/s41467-025-60786-2)
Supplement: Supplementary file 1 — Supplementary Information [file 41467_2025_60786_MOESM1_ESM.pdf]

Supporting Information

A Non-Pyrophoric Precursor for the Low Temperature Deposition of Metallic Aluminium

Erica N. Faria[1], Samuel P. Douglas[1], Shreya Mrig[1], Leonardo Santoni[1], Adam J. Clancy[1], Daniel W. N. Wilson[1], Caroline E. Knapp[1]*

[1]*Department of Chemistry, 20 Gordon Street, WC1H 0AJ, University College London, London (UK). Email: caroline.knapp@ucl.ac.uk*

Contents:

1. Results and discussion of compounds 1-5 [Al(R-acnac)$_2$Cl] (R = Me(1), Et (2), Ph (3), ^{i}Pr (4), Mes (5)) and [Li(12-crown-4][AlH$_2$(^{i}Pr-acnacH)AlH$_3$] (7).

2. Experimental details

3. Thermogravimetric Analysis (TGA)

4. Powder X-Ray Diffraction (PXRD)

5. X-Ray Photoelectron Spectroscopy (XPS)

6. Air- and moisture stability test of cluster 8 and dimethyl amine alane (DMEAA)

7. Transmission electron microscopy (TEM)

8. References

1. Results and discussion of compounds 1-5 [Al(R-acnac)$_2$Cl] (R = Me(1), Et (2), Ph (3), ^{i}Pr (4), Mes (5))

1.1. [Al(MeCN(Me)CHC=OMe)$_2$Cl] ([Al(Me-acnac)$_2$Cl], **1**)

The crystal structure of compound **1** was determined *via* SCXRD and is displayed in Figure S1. Compound **1** crystallises in the orthorhombic space group *Pbca*. The complex is a monomer containing two β-ketoiminate ligands and one chloride bound to one aluminium atom, in a mildly distorted trigonal bipyramidal arrangement around the metal. The chelating β-ketoiminate ligands bind in a bidentate mode, the Cl and O atoms occupy the equatorial positions while the N atoms occupy the axial positions. This motif is likely preferred for ligands with less bulky R groups, with equatorial bonds often being shorter, allowing a stronger binding interaction between Al and O, which exhibit high affinity to bond to one another due to the electronegativities of the elements of 1.61 and 3.44 respectively, in the Pauling electronegativity scale.[1]

The trigonal bipyramidal geometry is distorted with the two $AlOC_3N$ six-membered rings, angling away from the large Cl atom, with the N(1)–Al(1)–Cl(1) and N(2)–Al(1)–Cl(1) angles being 91.20(4)°and 91.63(3)°, respectively. There is also distortion between the axial and equatorial positions caused by steric constraints of the ligand, displayed by the pincer angles O(1)–Al(1)–N(1) and O(2)–Al(1)–N(2) of 90.90(4) and 91.33(4)°, respectively. Finally, there are distortions between equatorial atoms, with O(1)–Al(1)–Cl(1) and O(2)–Al(1)–Cl(1) angles of 118.66(3) and 117.09(4)°, respectively, which deviates from the expected 120° angle, most likely caused by the presence of the large electronegative Cl atom.

As expected, the Al–O bond lengths of 1.7850(9) and 1.7872(9) Å are shorter compared to the Al–N bond lengths of 1.9949(12) and 1.9915(11) Å, as a result of the smaller size and larger ionic contribution of the oxygen atoms. The Al–Cl bond is 2.2197(5) Å and is comparable with the few examples in the literature.[2–4] Bond lengths and angles of the two β-ketoiminate ligands vary only slightly and are, in most cases, within three estimated standard deviations of one another, hence, are crystallographically identical.

In addition to SCXRD characterisation, compound **1** was also identified *via* ^1H, $^{13}C\{^1H\}$ NMR, MS and EA. As expected, the NH proton resonance of the β-ketoimine pro-ligand is absent in the ^1H NMR spectrum of **1**, the doublet corresponding to the *N*-methyl peak has lost its multiplicity and all peaks are shifted compared to the pro-ligand. This shift is especially

apparent for the *N*-substituent CH_3 group, which drastically shifted downfield by 1.11 ppm, due to N electron donation to the Al centre and the resulting hyperconjugation. $^{13}C\{^1H\}$ NMR is in agreement with the ^1H NMR and also confirms the product.

1.2. $[Al(MeCN(Et)CHC{=}OMe)_2Cl]$ ($[Al(Et\text{-}acnac)_2Cl]$, **2**)

The crystal structure of compound **2** was determined *via* SCXRD and is displayed in Figure S1. Compound **2** crystallised in the lower symmetry triclinic space group *P–1*. Two bidentate ligands bind to the metal in the same mode as with compound **1** (R = Me). There are two independent molecules in the unit cell for complex **2** and, due to their similarity, only one is described here. A trigonal bipyramidal arrangement is also observed around the metal, with the N atoms bonded axially.

Overall, there are smaller distortions in the ethyl analogue compared to the methyl, displayed by the N(1)–Al(1)–N(2), O(1)–Al(1)–Cl(1) and O(2)–Al(1)–Cl(1) angles of 178.81(5), 120.61(4), and 117.30(4)° respectively. Al–O bonds are marginally shorter (Al(1)–O(1): 1.7839(10) Å, Al(1)–O(2): 1.7817(10) Å), whilst Al–N bonds are marginally longer (Al(1)–N(1): 2.0009(11) Å, Al(1)–N(2): 2.0051(11) Å), most likely due to the added size of the ethyl group and delocalised nature of the ligands. The Al(1)–Cl(1) bond of the ethyl complex (2.2150(5) Å) is slightly shorter than in the methyl complex, which may be as a result of greater N atom electron donating effects caused by greater hyperconjugation from the longer carbon chain supplying more electron density to the Al atom which can, in turn donate more electron density to the Cl atom, producing a shorter bond.

In addition to SCXRD characterisation, compound **2** was also identified *via* ^1H, ^{13}C NMR, MS and EA. As expected, the ^1H NMR spectrum of compound **2** does not show any resonances for the NH proton of the β-ketoimine pro-ligand, there is a loss of multiplicity at the *N*-adjacent methylene group and peaks are shifted. Interestingly, the complexation of this compound to the aluminium renders the methine proton diastereotopic and results in geminal coupling at the methylene, CH_2 groups, shown by the separation of the environment into two broad peaks at 3.80 and 3.41 ppm, in a 2:2 ratio. Coordination results in the proton environments becoming inequivalent. The methine proton is the most downfield peak, found at 4.81 ppm. The terminal protons for the keto and enamino groups on the backbone are observed at 1.78 and 1.47 ppm, respectively, both integrating to 6H each for the two ligands in the complex. The methyl protons at 1.20 ppm have been upfield shifted relative to **1**, due to being one

methylene group further away from the electron-withdrawing nitrogen atom. ^{13}C NMR environments are in agreement with the ^{1}H NMR.

1.3. [Al(MeCN(^{i}Pr)CHC=OMe)$_2$Cl] ([Al(^{i}Pr-acnac)$_2$Cl], **3**)

The crystal structure of compound **3** was determined *via* SCXRD (Figure S1) and it crystallised in the monoclinic space group *C*2/*c*. The two ligands bind to the metal in a bidentate mode, with the second ligand generated by symmetry *via* a twofold rotation axis that lies along the Al(1)–Cl(1) bond. The trigonal bipyramidal arrangement around the metal is distorted, with the N atoms bonding axially. The isopropyl analogue has marginally more distorted bond angles compared to the ethyl complex (**2**), with N(1)–Al(1)–N(1)′ and O(1)–Al(1)–Cl(1) of 178.34(8) and 119.37(4)° respectively, not being as distorted as the methyl analogue (**1**).
The O(1)–Al(1)–N(1) angle (92.03(5)°) departs the most from linearity compared the other synthesised structures of this motif, due the three-dimensional steric bulk of the isopropyl group, allowing it be further away from the adjacent backbone CH_3 group below it. Al–O (1.7720(11) Å) and Al–N (2.0259(12) Å) bonds follow the observed trends in increasing steric bulk of the *N*-substituent and there is a very marginal change in Al–Cl bond distance from the methyl and ethyl complexes (Al(1)–Cl(1): 2.2132(8) Å).

In addition to SCXRD characterisation, compound **3** was also identified *via* ^{1}H, ^{13}C{^{1}H} NMR, MS and EA. As expected, the ^{1}H NMR spectrum of **3** does not show any resonances for the NH proton of the β-ketoimine pro-ligand, indicating coordination. There is a loss of multiplicity at the *N*-adjacent methylene group and peaks are shifted. Multiplicity is not resolved at the terminal methyl groups on the isopropyl R group. The peak corresponding to the methine proton on the ligand backbone is found at 4.75 ppm, closely followed by the methine proton adjacent to the N atom. The remaining peaks correspond to the terminal protons for the keto (1.76 ppm) and enamino (1.65 ppm) groups on the backbone, as well as the methyl groups of the isopropyl group (1.41 ppm). ^{13}C{^{1}H} NMR environments are in agreement with the ^{1}H NMR, showing non-equivalent terminal methyl groups on the isopropyl group.

1.4. [Al(MeCN(Ph)CHC=OMe)$_2$Cl] ([Al(Ph-acnac)$_2$Cl], **4**)

The crystal structure of compound **4** was determined *via* SCXRD and is displayed in Figure S1. Compound **4** crystallised in the monoclinic space group *C2/c*. Two bidentate ligands bind to the metal in a bidentate mode with the second ligand generated by symmetry *via* a twofold rotation axis that lies along the Al(1)–Cl(1) bond. The trigonal bipyramidal arrangement

around the metal is the most distorted from the idealised geometry, with the N atoms still bonded axially. The axial N(1)–Al(1)–N(1)′ angle of 172.99(6)° in **4** (compared to 178.34(8)° in **3**) indicates the ligands bend further away from the Cl atom and may be a result of the orientation of the phenyl groups, as such to lower steric clash between the ligands. The O(1)–Al(1)–N(1) pincer angle of 90.16(4)° is the lowest among compounds **1**-**5**, most likely due to the low axial N–Al–N angle. There is a small difference in the Al–O bond distance compared to the isopropyl analogue (1.7720(11) Å in **3** and 1.7718(9) Å in **4**), however there is a noticeable difference in the Al–N bond distance (**3**: 2.0259(12) Å; **4**: 2.0377(11) Å) with the longest axial Al–N bond length, due to the bulkier nature of the phenyl group. The longest axial Al–N bond length, results in the shortest Al–Cl length of 2.1896(7) Å.

In addition to SCXRD characterisation, compound **4** was also identified *via* ^{1}H, $^{13}C\{^{1}H\}$ NMR, MS and EA. Compared to the ^{1}H NMR spectrum of the pro-ligand, the peaks are shifted and resonance corresponding to the NH is not present. Singlet peaks at 4.88 ppm (C*H*), 1.47 (CH_3CO), and 1.36 ppm (CH_3CN) in a 2:6:6 ratio agree with the formation of **4**. The aromatic region in the ^{1}H NMR is difficult to fully characterise, due to their being broad overlapping peaks, as well as the NMR solvent residual peak of C_6D_6 at 7.16 ppm occurring in the same region, however the overall integration of this region sums to ten protons. $^{13}C\{^{1}H\}$ NMR is consistent with the ^{1}H NMR.

1.5. [Al(MeCN(Mes)CHC=OMe)$_2$Cl] ([Al(Mes-acnac)$_2$Cl], **5**)

The crystal structure of **5** was determined *via* SCXRD and is displayed in Figure S1. Complex **5** crystallises in the tetragonal space group $P4_32_12$, with the two ligands also binding to the metal in a bidentate mode and the second ligand is generated by symmetry *via* a two-fold rotation axis that lies along the Al(1)–Cl(1) bond. In this case, and unlike compounds **1**-**4**, the O atoms occupy the axial positions of the trigonal bipyramidal geometry instead of the N atoms, with an O(1)–Al(1)–O(1)′ angle of 179.77(9)°, deviating only slightly from the linearity of 180°. Similar to the axial angle, the equatorial angles (N(1)–Al(1)–N(1)′: 119.82(8)°, N(1)–Al(1)–Cl(1): 120.09(4)°) deviate minimally from linearity. Comprehensibly, the Al–O bond length has increased (1.8286(12) Å) while the Al–N bond has decreased (1.9718(13) Å) compared to the complexes **1**-**4**, due to the tendency of axial bonds being longer than equatorial bonds. The Al–Cl bond length of 2.2176(8) Å is comparable to all other four structures, apart from the phenyl analogue, which is shorter.

In addition to SCXRD characterisation, compound **5** was also identified *via* ^{1}H, ^{13}C{^{1}H} NMR, MS and EA. The ^{1}H NMR spectrum of compound **5** does not show any resonances for the NH proton of the β-ketoimine pro-ligand, indicating ligand coordination, with all peaks shifted relative to the β-ketoimine pro-ligand. Complexation results in a splitting of the aryl protons Ar−H (6.84, 6.75 ppm) and *ortho* Ar−CH_3 (2.52, 2.00 ppm) signals, suggesting groups are no longer equivalent due to their inability to rotate about the N–C bond as a result of steric encumbrance. ^{13}C{^{1}H} NMR environments are in agreement with the formation of **5**.

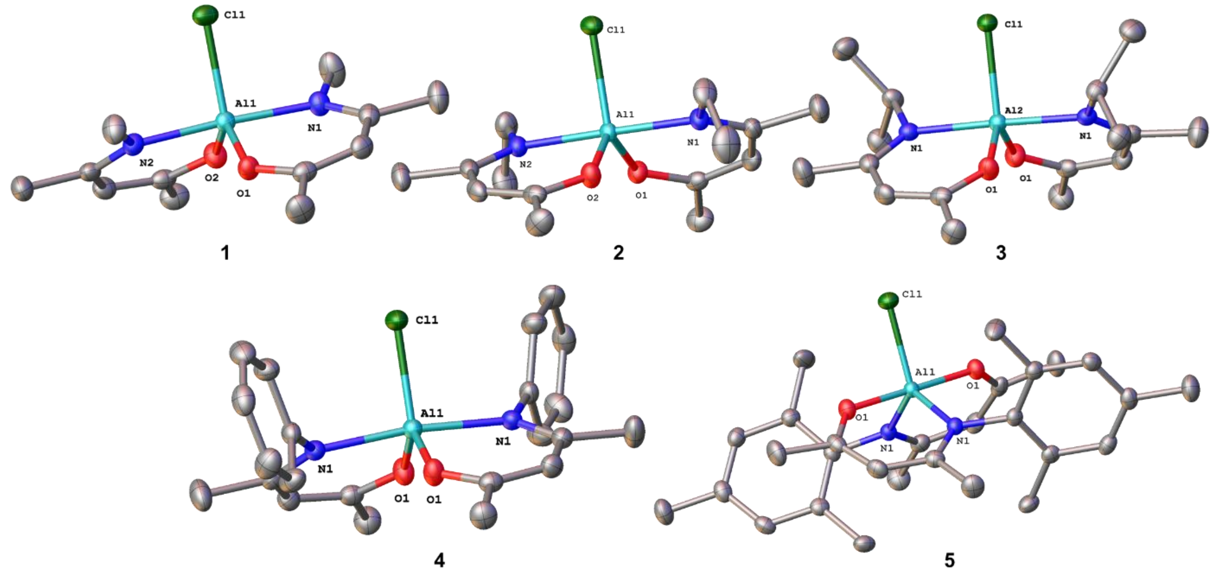

Figure S1: *Molecular structures of compounds Al(R-acnac)$_2$Cl], where R = Me (**1**), Et (**2**), ^{i}Pr (**3**), Ph (**4**) and Mes (**5**). Thermal ellipsoids are drawn at 50% probability and hydrogen atoms are omitted for clarity.*

1.6. Li[AlH_2(^{i}Pr-Hacnac)AlH_3]$_n$ (**6**)

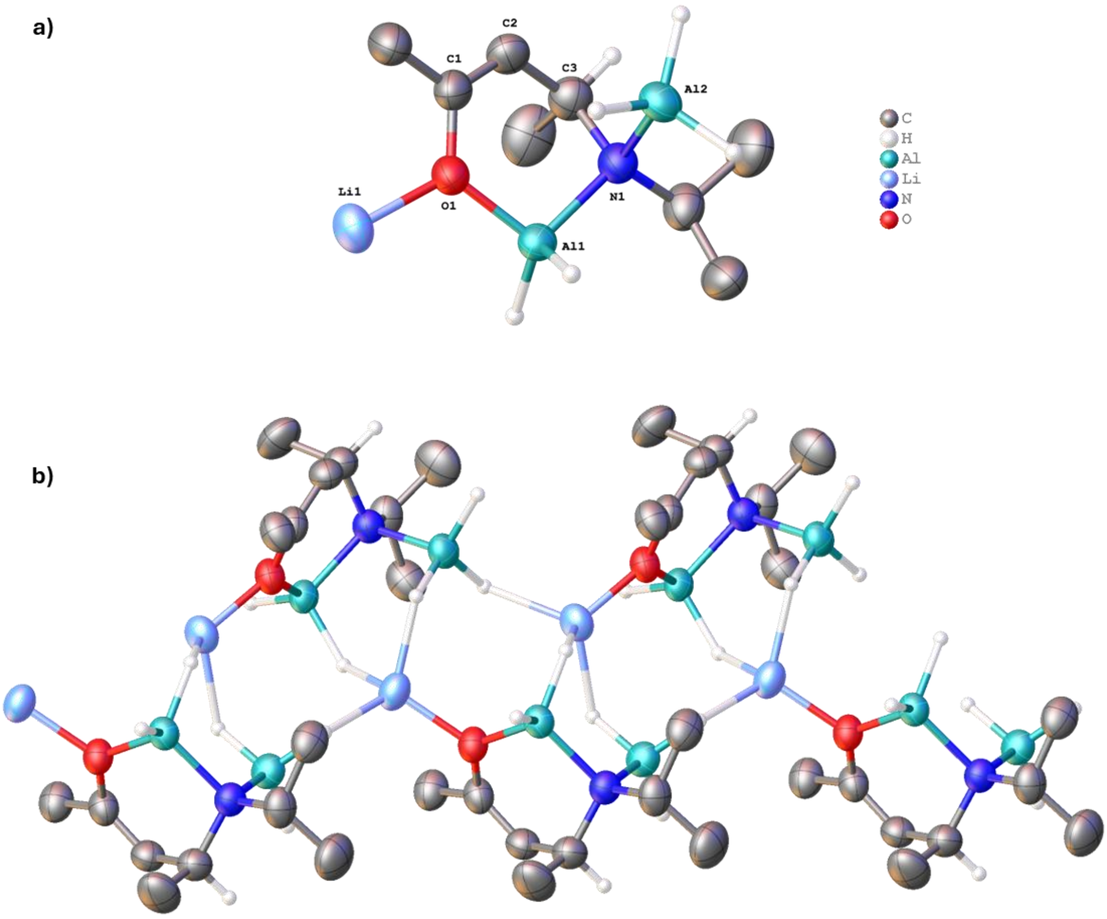

Figure S2: *a) Repeating unit of **6**; b) Extended polymeric structure of Li[AlH_2(^{i}Pr-Hacnac)AlH_3]$_n$ (**6**). Thermal ellipsoids pictured at 50% probability. Some hydrogen atoms omitted for clarity. Selected bond lengths (Å): O1–Li1: 1.914(9); O1–Al1: 1.786(4); O1–C1: 1.376(6); C1–C2: 1.327(7); C2–C3: 1.502(7); C3–N1: 1.521(7); N1–Al1: 1.937(4); N1–Al2: 1.969(4).*

1.7. [Li(12-crown-4)][AlH_2(^{i}Pr-Hacnac)AlH_3] (**7**)

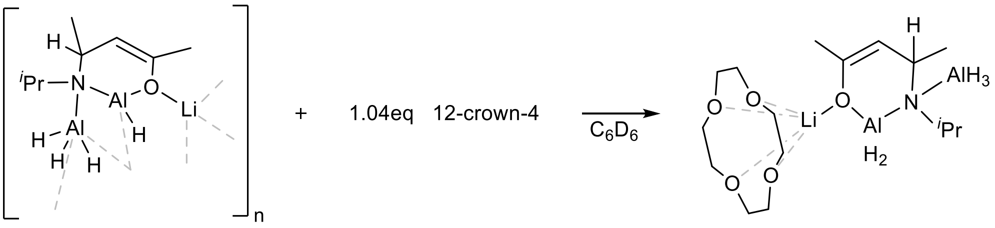

Figure S3: *Reaction of one equivalent of the polymer Li[AlH_2(R-Hacnac)AlH_3]$_n$ (**6**) with a slight excess of 12-crown-4 to isolate the corresponding monomeric structure [Li(12-crown-4)][AlH_2(^{i}Pr-Hacnac)AlH_3] (**7**).*

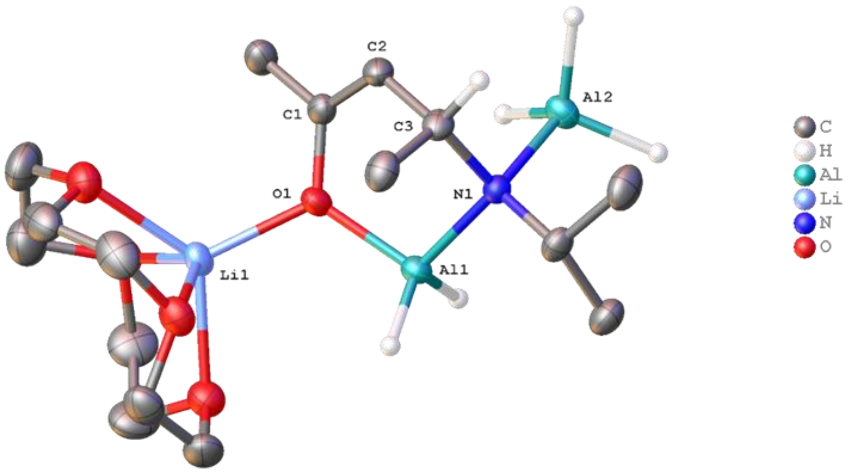

Figure S4: *Molecular structure of [Li(12-crown-4)][AlH_2(^{i}Pr-Hacnac)AlH_3] (**7**). Thermal ellipsoids pictured at 50% probability. Some hydrogen atoms omitted for clarity. Selected bond lengths (Å): O1–Li1: 1.937(5); O1–Al1: 1.795(2); O1–C1: 1.373(3); C1–C2: 1.333(4); C2–C3: 1.504(4); C3–N1: 1.523(4); N1–Al1: 1.952(2); N1–Al2: 1.993(2).*

Compound **7** crystallises in the orthorhombic space group $P2_12_12_1$. The structure is a monomer featuring one chelating bidentate β-ketoiminate ligand bonding to one aluminium atom *via* both oxygen and nitrogen atoms and the backbone of the ligand remains protonated compared to the polymeric structure **6**, therefore rendering the ligand not planar due to the presence of the chiral C3 carbon. The nitrogen atom remains additionally coordinated to a second aluminium that is now terminal, and the oxygen atom also remains bound to a lithium atom, but the latter now sits isolated in the electron-rich cavity of the 12-crown-4 sequestering agent. Both aluminium atoms adopt a tetrahedral geometry.

In comparison to the extended structure of **6**, a slight lengthening of the O1–Al1 bond (1.795(2) for **7** and 1.786(4) for **6**) is observed in the isolated monomer. A slight lengthening is also observed for the O1–Li1 and N1–Al2 bonds (1.937(5) and 1.993(2) in **7** compared to 1.914(9) and 1.969(4) for **6**) while all other bonds remained similar.

1.8. $[AlH_2AlH_2(N–Mes)_3(AlH_2 \cdot Li(Et_2O)_2)_2]$ (**8**)

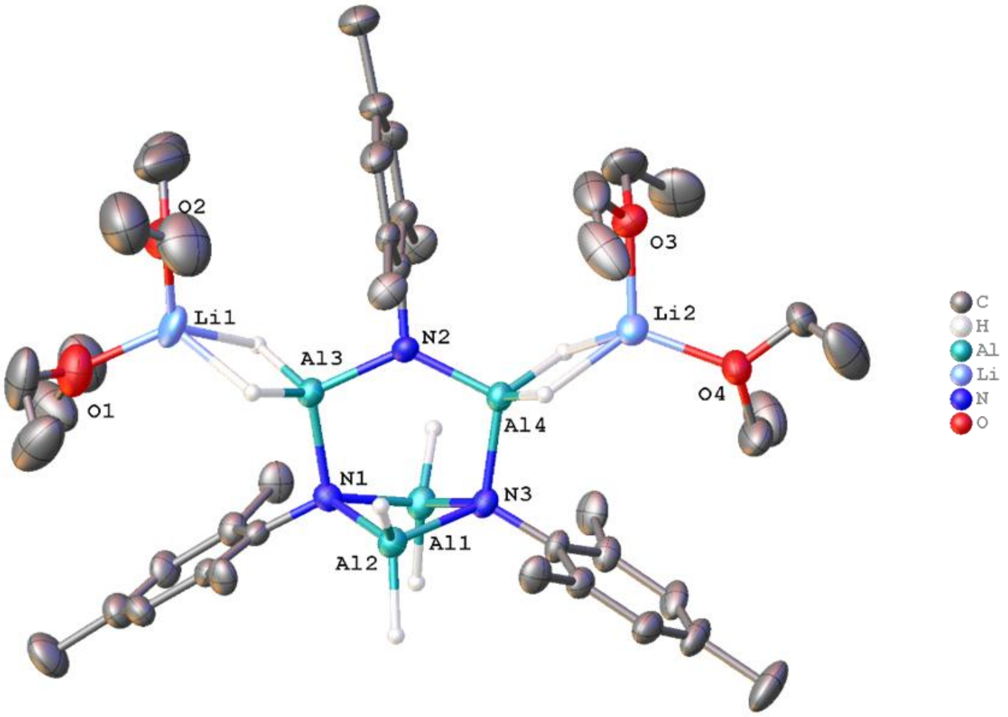

***Figure S5**: Molecular structure of $[AlH_2AlH_2(N–Mes)_3(AlH_2 \cdot Li(Et_2O)_2)_2]$ (**8**). Thermal ellipsoids pictured at 50% probability. Some hydrogen atoms omitted for clarity. Selected bond lengths (Å) and angles (deg): Al1–N1: 1.963(4); Al1–N3: 1.963(4); Al2–N1 1.977(4); Al2–N3: 1.956(4); N1–Al3: 1.902(4); Al3–N2: 1.815(4); N2–Al4: 1.816(4); Al4–N3: 1.903(4); Al3–N2–Al4: 128.7(2); Al3–N2–C10: 111.4(3); Al4–N2–C10 119.9(3).*

***Note:** Crystallographic data for the structures reported have been deposited at the Cambridge Crystallographic Data Centre, under deposition numbers CCDC 2405550-2405557. Copies of the data can be obtained free of charge via https://www.ccdc.cam.ac.uk/structures/.*

2. Experimental details

2.1. General experimental details

All reactions and product manipulations were performed using standard Schlenk-line and glovebox techniques, both using N_2 as the inert gas. All glassware used in air-sensitive reactions were flamed dried prior to use.

All reagents were procured from Sigma Aldrich/Merck and used as received unless otherwise stated. All non-deuterated solvents were stored under N_2 in gas-tight ampoules over activated molecular sieves (3 or 4 Å).[5] Deuterated solvents were obtained from Cambridge Isotope Laboratories or Sigma Aldrich/Merck and were degassed before use and stored over 3 Å molecular sieves. Amines were used as received for ligand synthesis and the β-ketoiminate ligands were synthesised according to previously reported literature from the group.[6] $LiAlH_4$ powder, reagent grade 95%, was procured from Sigma Aldrich and purified by dissolution and recrystallisation from diethyl ether using a literature procedure.[7]

NMR spectroscopy: NMR samples were prepared inside an inert atmosphere glovebox in NMR tubes fitted with a gas-tight valve when necessary. ^1H and $^{13}C\{^1H\}$ NMR spectra were collected on Bruker 400, 500 or 600 MHz NMR instruments at 298 K. ^1H and $^{13}C\{^1H\}$ NMR spectra are reported relative to tetramethylsilane (TMS) and referenced to the most downfield residual solvent resonance where possible (*d*$_6$-benzene (C_6D_6): δ_H = 7.16 ppm, δ_C = 128.06 ppm). ^1H and $^{13}C\{^1H\}$ NMR assignments were confirmed by ^1H–^1H (COSY) and ^1H–^{13}C (HSQC and HMBC) experiments where necessary. Data analysis was performed using either MestreNova or TopSpin software.

Elemental Analysis (CHN): used to determine the percentage of C, H and N in each sample using a Carlo Erba CE1108 Elemental Analyser (London Metropolitan University). Samples were submitted in a vial inside a vial prepared in the glovebox. We note that due to the desired facile decomposition of the compounds, elemental composition is thought to vary from other techniques due to partial decomposition before/during elemental analysis.

Mass spectroscopy: compounds were dissolved in dry toluene, ionised using electrospray ionisation and detected in positive (ESI+) or negative mode (ESI-) on a Waters LCT Premier XE.

Thermogravimetric Analysis (TGA): Measurements were made using a PerkinElmer STA6000 TGA. The instrument had a sensitivity of 0.1 mg and used N_2 as shield gas. Samples were prepared in a glovebox, where they were placed in 85 μL aluminium pans and sealed. The pans were pierced just before measurement to allow for mass loss. The samples were heated from 30 °C (room temperature) up to a maximum of 500 °C, at a heating rate of 5−10 °C min^{-1} and under flow of shield gas (N_2).

Infrared Spectroscopy: ATR-IR spectra were recorded on a Shimadzu IRAffinity-1S Fourier Transform Infrared Spectrophotometer.

Single crystal X-ray diffraction (SCXRD): Single crystal X-ray diffraction data was collected using a SuperNova Atlas (Dual) diffractometer. Suitable crystals were selected under Fomblin oil and mounted on a nylon loop. Data was collected at 150 K, using Cu K*α* radiation (*λ* = 1.54184 Å). Structures were solved using Olex2 software.[8] The structure of compounds **1**-**4** and **7** was solved with the olex2.solve[9] structure solution program using Charge Flipping and refined with the olex2.refine[9] refinement package using Gauss-Newton minimisation. The structure of **5** was solved with the olex2.solve[9] structure solution program using Charge Flipping and refined with the ShelXL[10] refinement package using Least Squares minimisation. **6** the structure was solved with the Unknown [2] structure solution program using Unknown and refined with the ShelXL[10] refinement package using Least Squares minimisation. **8** was solved with the ShelXT [11] structure solution program using Intrinsic Phasing and refined with the ShelXL [10] refinement package using Least Squares minimisation. Crystallographic details for **1**-**8** are available from the CSD (www.ccdc.cam.ac.uk/data_request/cif) numbers: CSD-2405550-2405557

Powder (P)XRD - samples were loaded in 0.5 mm glass capillaries, measurements were performed on a STOE XRD using either a Cu kα or Mo kα X-ray source , on a rotating sample (~5 Hz) in 2θ 0.2° steps for 10s at each step. Grazing Incident (GI)XRD: Patterns were recorded using an Empyrean X-raydiffract ion system with Cu Kα1 (λ = 1.54056 Å) and Cu Kα2 (λ = 1.54439 Å). The GIXRD patterns were collected with a scanning rate of 0.05° s^{-1} over a 2θ scan from 10° to 80°.

Scanning Electron Microscopy: Side-on images of the aluminium film on glass were obtained using a JEOL JSM-7600F field emission SEM instrument. All samples were coated with a layer of gold prior to imaging.

Conductivity Measurements: Sheet resistance measurements were carried out using an Ossila T2001A Four Point Probe (1.27 µm inter-probe separation). Measurements were taken at 3 different sites and averaged to give a mean and standard error.

2.2. Synthesis of aluminium compounds 1 – 8.

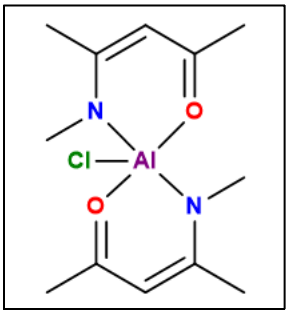

[Al(MeCN(Me)CHC=OMe)$_2$Cl] ([Al(Me-acnac)$_2$Cl], **1**)

LiHMDS (1.02 g, 6.10 mmol) in toluene (20 mL) was added to a solution of Me-acnacH (0.68 g, 6.01 mmol) in toluene (20 mL) at −78 °C. The reaction mixture was stirred at room temperature overnight to form a transparent, pale-yellow solution. The volatiles were removed under vacuo to produce a pale-yellow solid, which was re-dissolved in toluene (20 mL) and added to a slurry of $AlCl_3$ (0.40 g, 3.00 mmol) in 20 mL toluene at −78 °C. The reaction mixture was stirred at room temperature overnight to form a cloudy, pale-yellow solution, which was filtered, and the clear pale-yellow filtrate concentrated and stored at −20 °C. Overnight, white crystals suitable for single crystal X-ray measurement precipitated. The crystals were separated from the supernatant solution and dried under reduced pressure. Yield: 0.82 g (95%).

^{1}H NMR (500 MHz, C_6D_6) δ 4.81 (s, 2H, CH), 3.11 (s, 6H, CH_3), 1.79 (s, 6H, CH_3CO), 1.30 (s, 6H, CH_3CN).

$^{13}C\{^{1}H\}$ NMR (C_6D_6, 126 MHz) δ 173.92 (CH_3CO), 171.80 (CH_3CN), 100.57 (CH), 37.31 (CH_3), 24.82 (CH_3CO), 20.91 (CH_3CN).

MS: m/z [ESI+] 251 $[M – Cl]^+$.

CHN: Found (Calcd.) for $C_{12}H_{20}O_2N_2AlCl$: 50.11 (50.27), 7.07 (7.03), 9.50 (9.77).

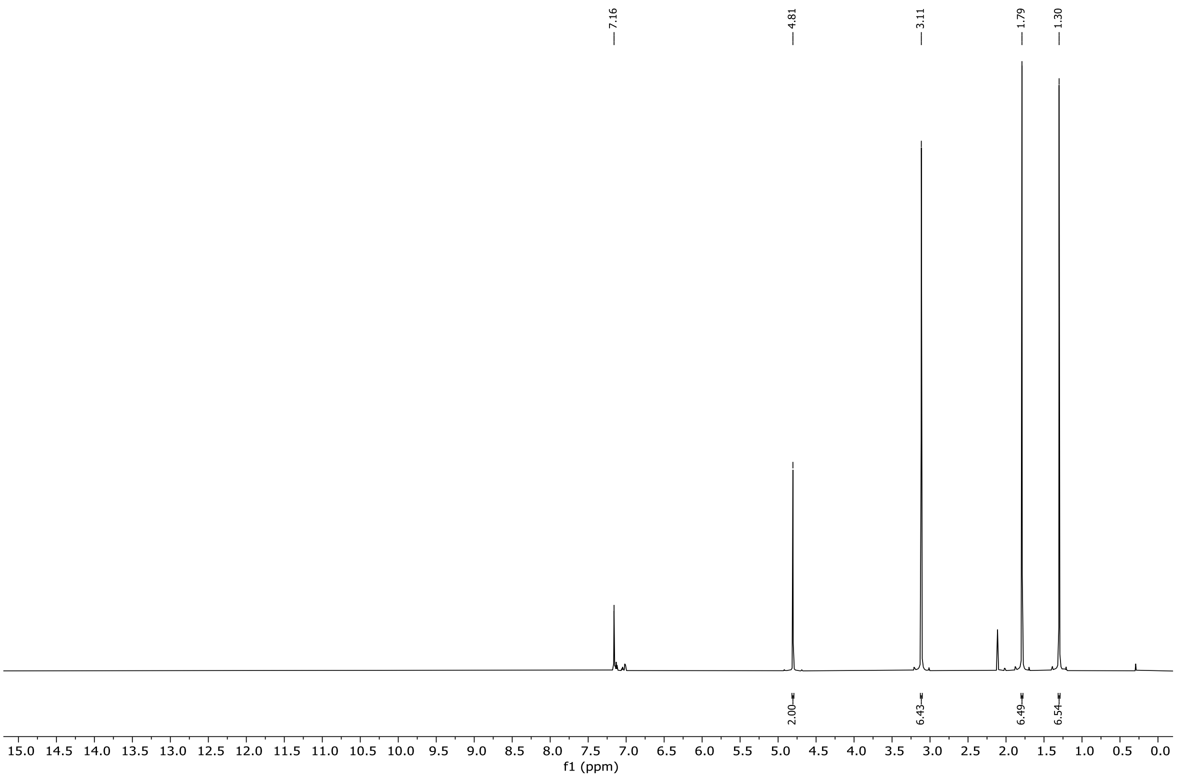

Figure S6: ^{1}H NMR (500 MHz) spectrum of a C_6D_6 solution of (**[Al(Me-acnac)$_2$Cl]**, (**1**).

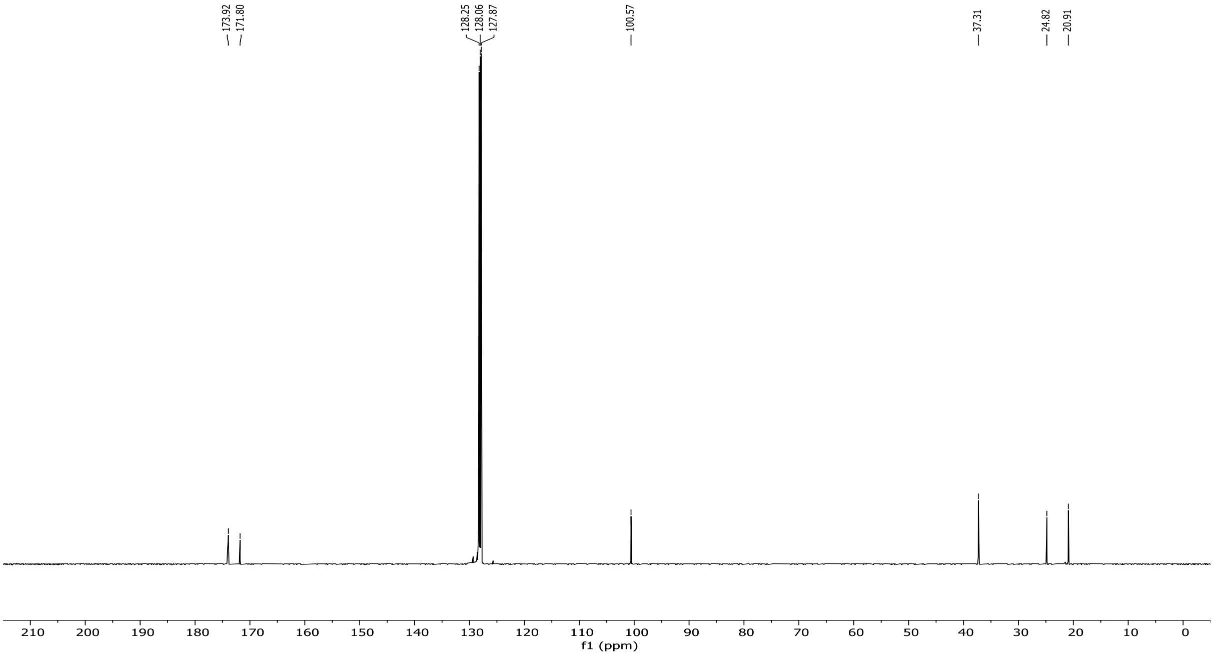

Figure S7: $^{13}C\{^{1}H\}$ NMR (126 MHz) spectrum of a C_6D_6 solution of (**[Al(Me-acnac)$_2$Cl]**, (**1**).

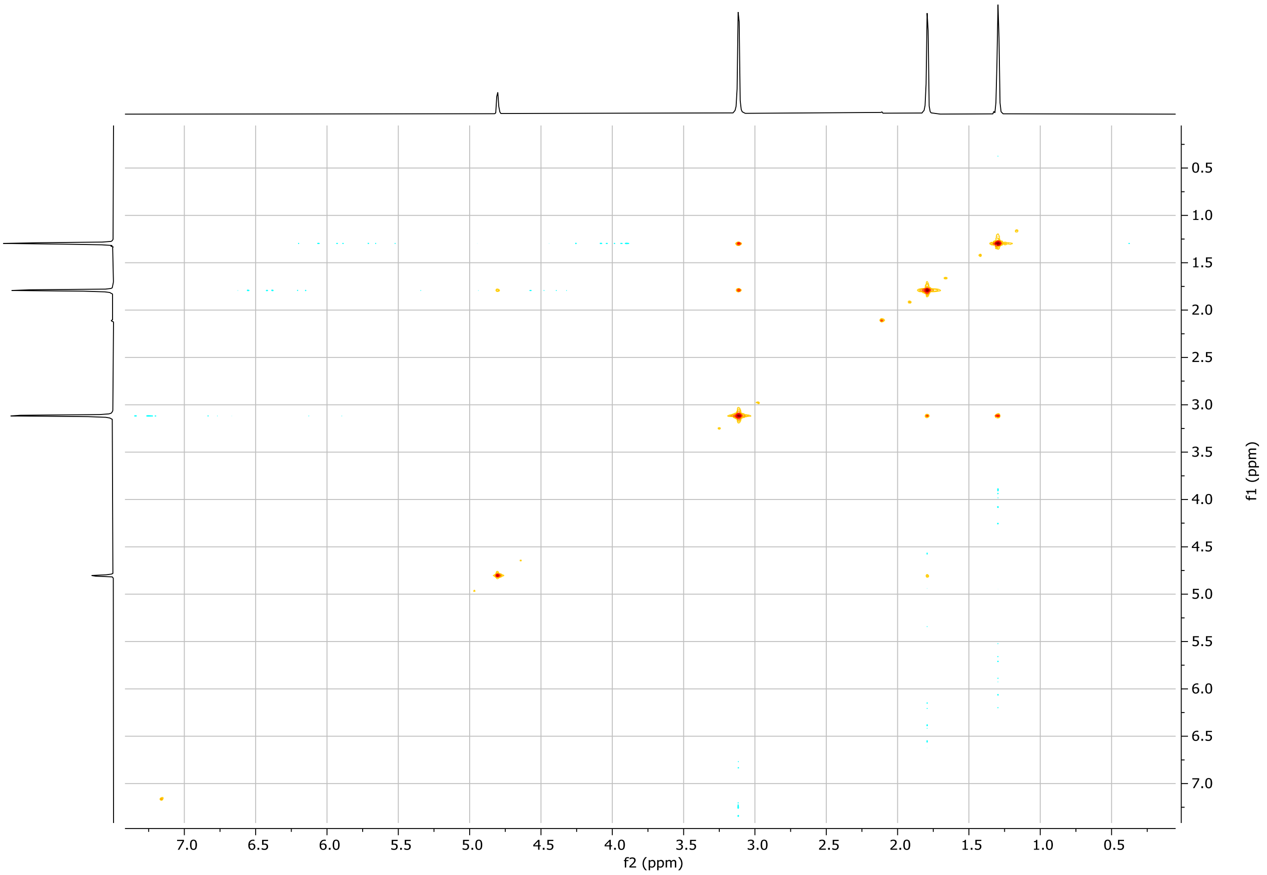

Figure S8: COSY NMR spectrum of a C_6D_6 solution of (**[Al(Me-acnac)$_2$Cl]**, (**1**).

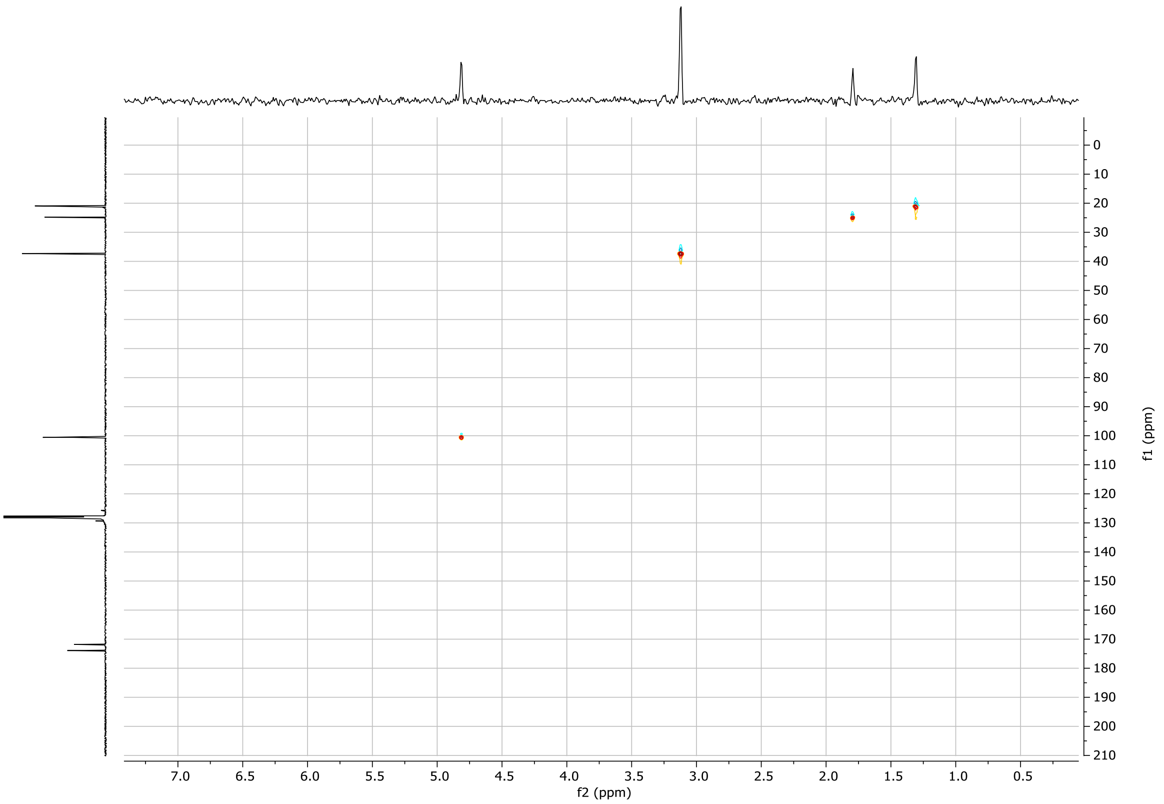

Figure S9: HSQC NMR spectrum of a C_6D_6 solution of (**[Al(Me-acnac)$_2$Cl]**, (**1**).

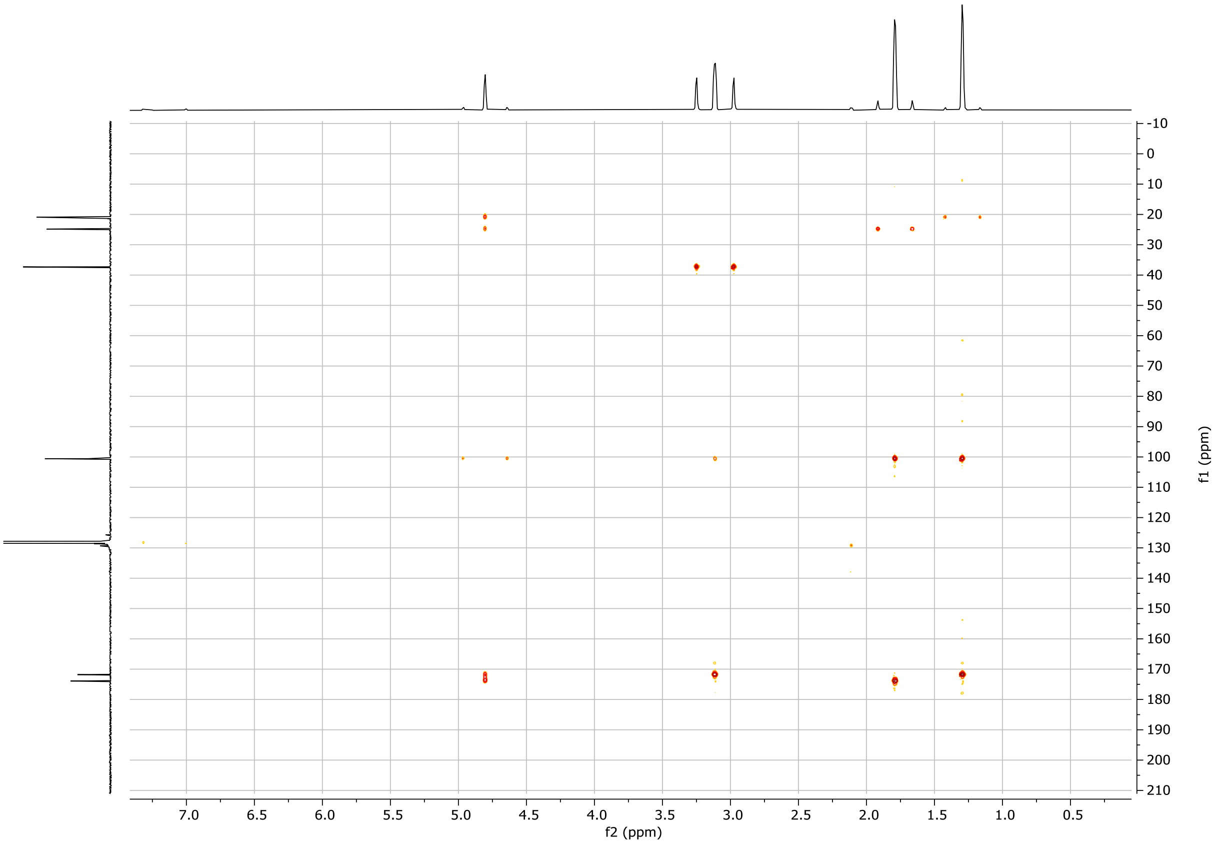

Figure S10: HMBC NMR spectrum of a C_6D_6 solution of (**[Al(Me-acnac)$_2$Cl]**, (**1**).

[Al(MeCN(Et)CHC=OMe)$_2$Cl] ([Al(Et-acnac)$_2$Cl], **2**)

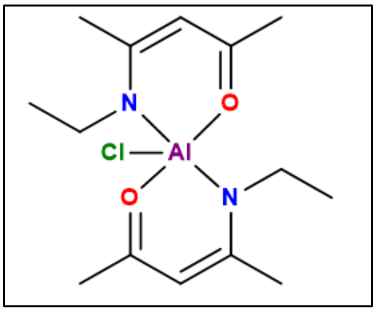

LiHMDS (1.00 g, 5.98 mmol) in toluene (20 mL) was added to a solution of Et-acnacH (0.77 g, 6.05 mmol) in toluene (20 mL) at −78 °C. The reaction mixture was stirred at room temperature overnight to form a transparent, pale-yellow solution. The volatiles were removed in vacuo to produce a pale-yellow solid, which was re-dissolved in toluene (20 mL) and added to a slurry of $AlCl_3$ (0.40 g, 3.00 mmol) in toluene (20 mL) at −78 °C. The reaction mixture was stirred at room temperature overnight to form a cloudy, pale-yellow solution, which was filtered to produce a clear solution of the same colour. The solvent was removed in vacuo to yield a pale-yellow solid, which was recrystallised from hexane and toluene overnight at −20 °C. White crystals suitable for single crystal X-ray measurement precipitated. The crystals were separated from the supernatant solution and dried under reduced pressure. Yield: 0.79 g (84%).

^{1}H NMR (C_6D_6, 500 MHz) δ 4.81 (2H, s, CH), 3.88 (2H, s, CH_2), 3.41 (2H, s, CH_2), 1.78 (6H, s, CH_3CO), 1.47 (6H, s, CH_3CN), 1.20 (6H, t, J = 7.1 Hz, CH_3).

^{13}C{^{1}H} NMR (C_6D_6, 126 MHz) δ 173.33 (CH_3CO), 171.37 (CH_3CN), 100.99 (CH), 44.30 (CH_2), 24.60 (CH_3CO), 20.34 (CH_3CN), 15.30 (CH_3).

MS: m/z [ESI+] 315 $[M]^+$, 279 $[M – Cl]^+$.

CHN: Found (Calcd.) for $C_{14}H_{24}O_2N_2AlCl$: 52.95 (53.42), 7.72 (7.68), 8.72 (8.90).

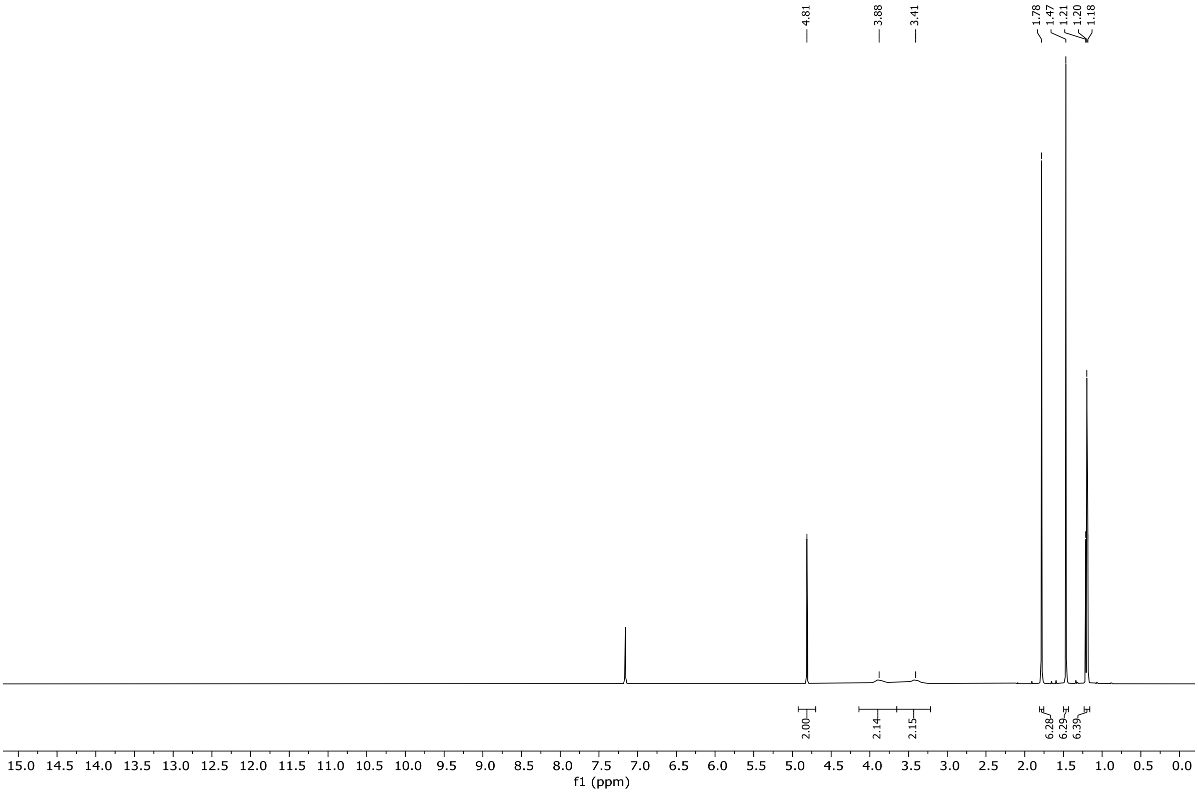

Figure S11: ^{1}H NMR (500 MHz) spectrum of a C_6D_6 solution of (**[Al(Et-acnac)$_2$Cl]**, (**2**).

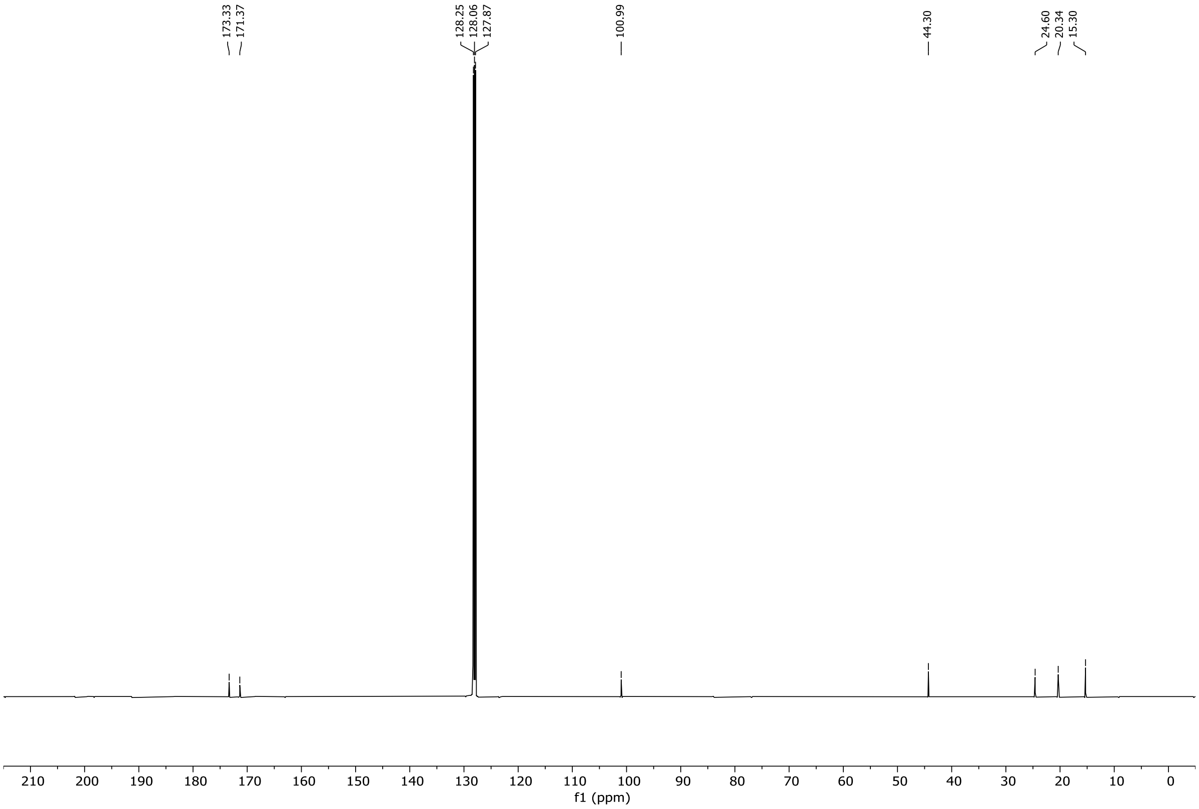

Figure S12: $^{13}C\{^{1}H\}$ NMR (126 MHz) spectrum of a C_6D_6 solution of (**[Al(Et-acnac)$_2$Cl]**, (**2**).

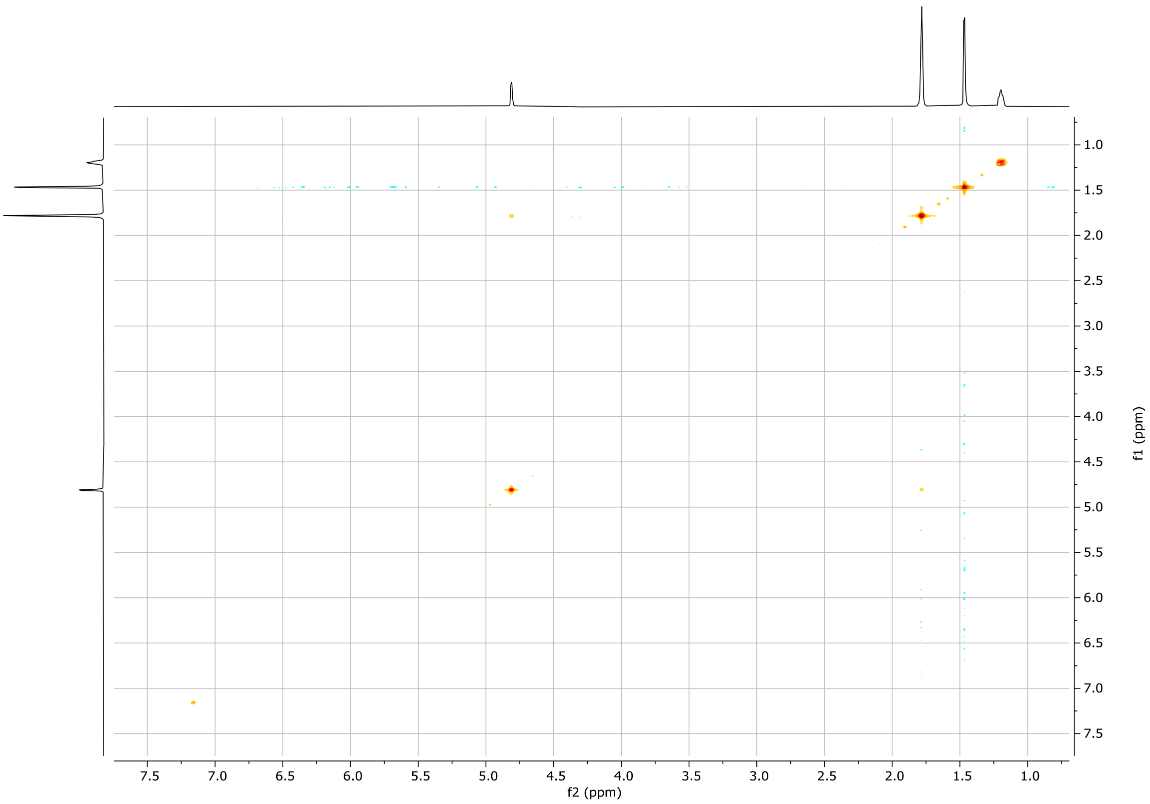

Figure S13: COSY NMR spectrum of a C_6D_6 solution of (**[Al(Et-acnac)$_2$Cl]**, (**2**).

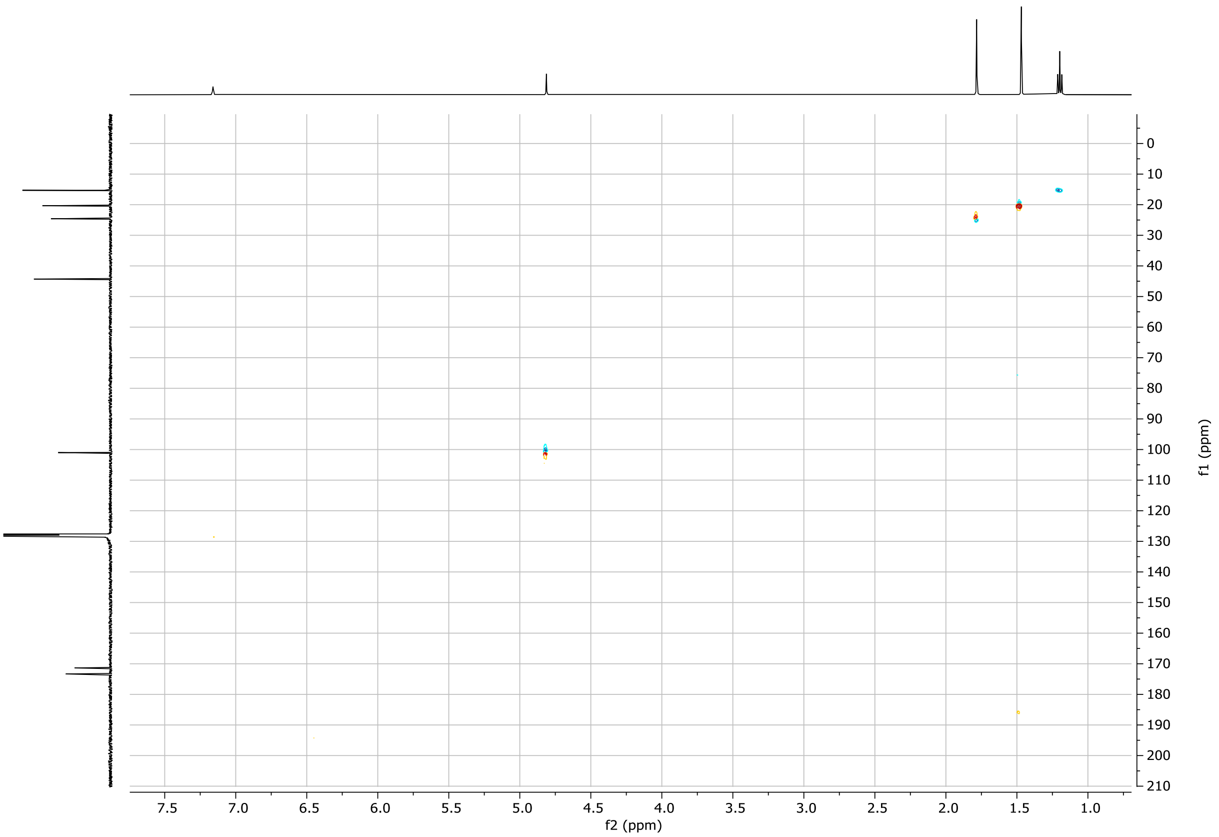

Figure S14: HSQC NMR spectrum of a C_6D_6 solution of (**[Al(Et-acnac)$_2$Cl]**, (**2**).

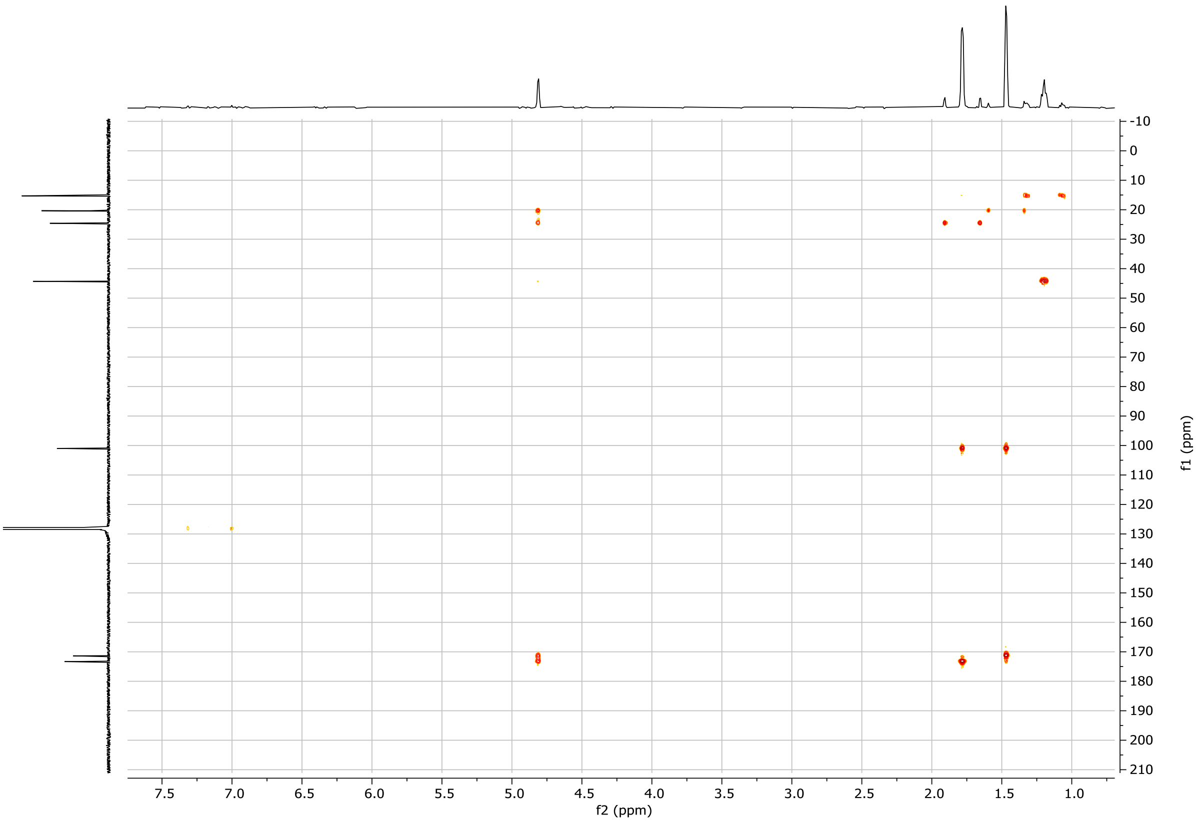

Figure S15: HMBC NMR spectrum of a C_6D_6 solution of (**[Al(Et-acnac)$_2$Cl]**, (**2**).

[Al(MeCN(^{i}Pr)CHC=OMe)$_2$Cl] ([Al(^{i}Pr-acnac)$_2$Cl], **3**)

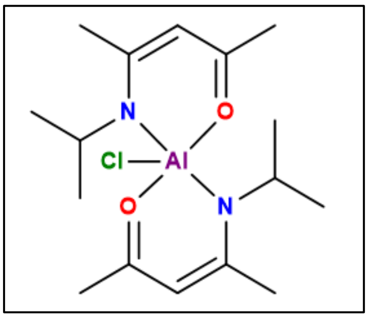

LiHMDS (0.58 g, 3.47 mmol) in toluene (20 mL) was added to a solution of ^{i}Pr-acnacH (0.54 g, 3.85 mmol) in toluene (20 mL) at −78 °C. The reaction mixture was stirred at room temperature overnight to form a cloudy, pale-yellow solution. The volatiles were removed in vacuo to produce a pale-yellow solid, which was redissolved in toluene (20 mL) and added to a slurry of $AlCl_3$ (0.23 g, 1.72 mmol) in toluene (20 mL) at −78 °C. The reaction mixture was stirred at room temperature overnight to form a cloudy, pale-yellow solution, which was filtered, with the clear pale-yellow filtrate concentrated and stored at −20 °C. Overnight, white crystals suitable for single crystal X-ray measurement precipitated. The crystals were separated from the supernatant solution and dried under reduced pressure. Yield: 0.51 g (86%).

^{1}H NMR (C_6D_6, 600 MHz) δ 4.75 (s, 2H, CH), 4.57 (br s, 2H, CH(CH_3)2), 1.76 (s, 6H, CH_3CO), 1.65 (s, 6H, CH_3CN), 1.41 (br s, 12H, CH_3).

^{13}C{^{1}H} NMR (C_6D_6, 151 MHz) δ 171.90 (CH_3CO), 170.58 (CH_3CN), 101.77 (CH), 51.26 (CH(CH_3)2), 24.52 (CH_3CO), 23.66 (CH_3CN), 22.47, 21.85 (CH_3).

MS: m/z [ESI+] 283 [M – Al – Cl + 3H]$^+$.

CHN: Found (Calcd.) for $C_{16}H_{28}O_2N_2AlCl$: 55.85 (56.05), 8.33 (8.23), 8.12 (8.17).

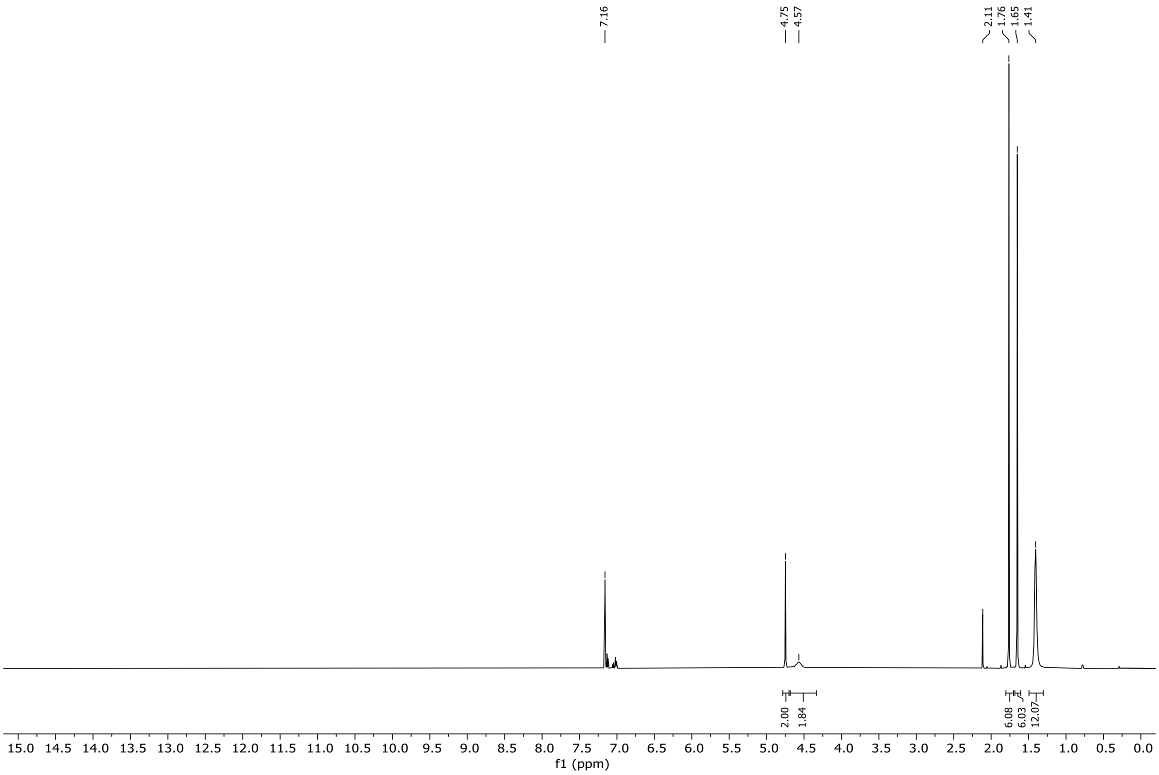

Figure S16: ^{1}H NMR (600 MHz) spectrum of a C_6D_6 solution of (**[Al(^{i}Pr-acnac)$_2$Cl]**, (**3**).

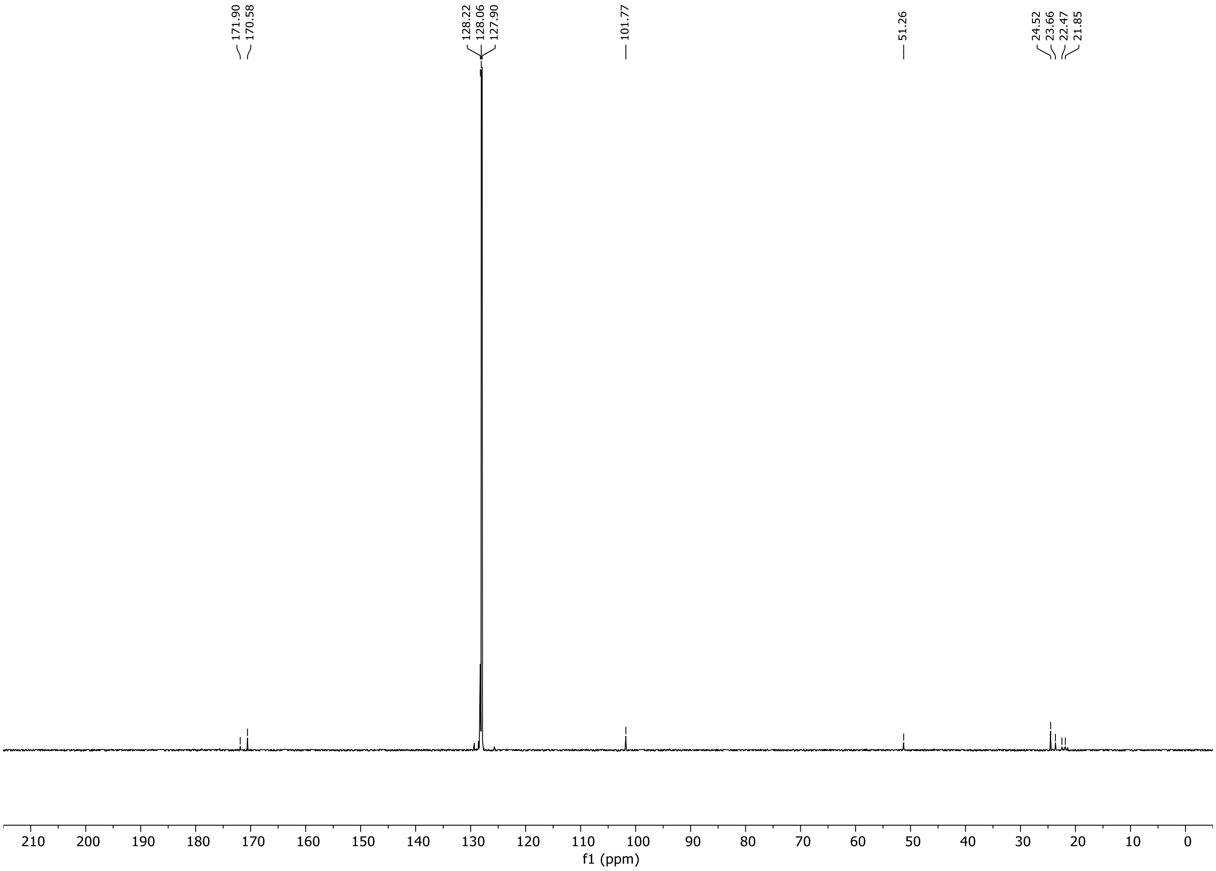

Figure S17: $^{13}C\{^1H\}$ NMR (151 MHz) spectrum of a C_6D_6 solution of (**[Al(^{i}Pr-acnac)$_2$Cl]**, (**3**).

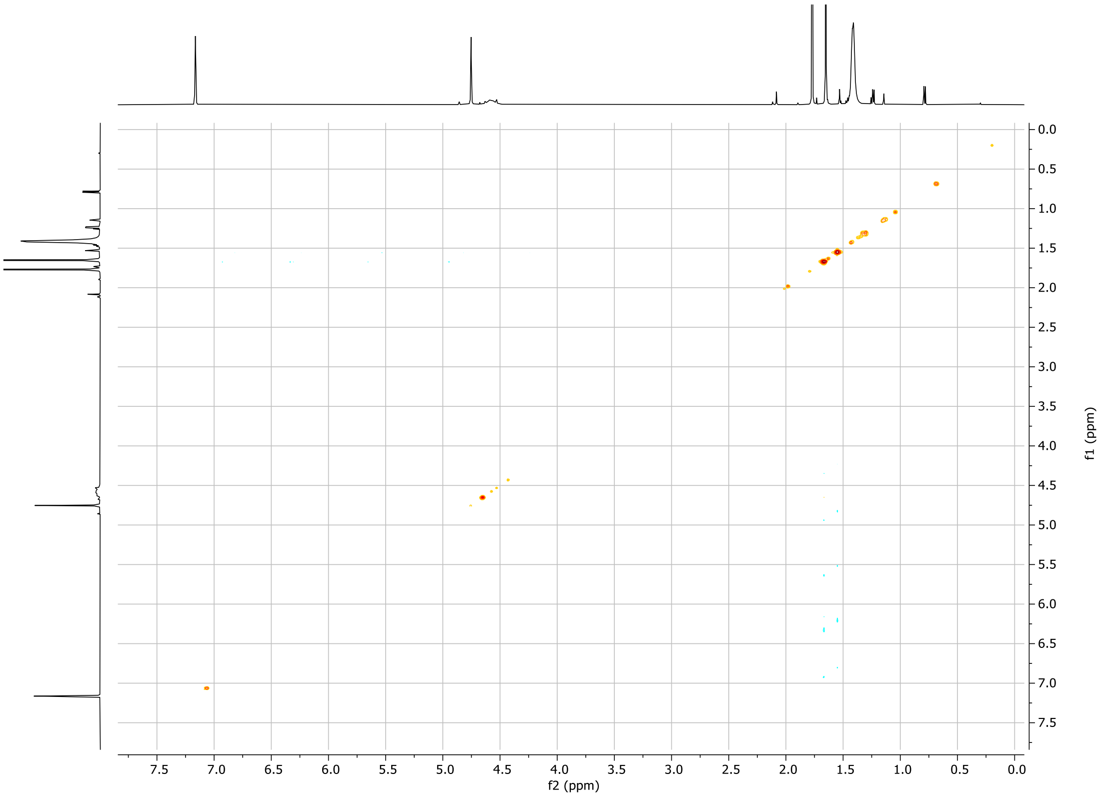

Figure S18: COSY NMR spectrum of a C_6D_6 solution of (**[Al(^{i}Pr-acnac)$_2$Cl]**, (**3**).

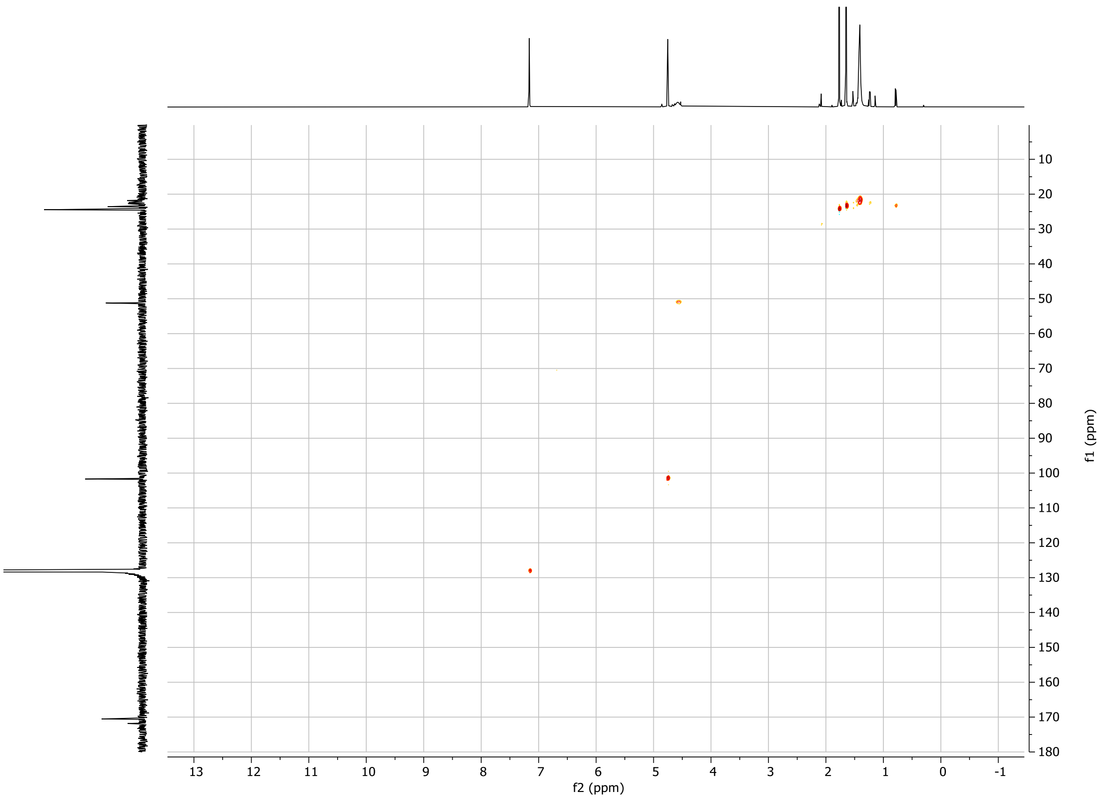

Figure S19: HSQC NMR spectrum of a C_6D_6 solution of (**[Al(^{i}Pr-acnac)$_2$Cl]**, (**3**).

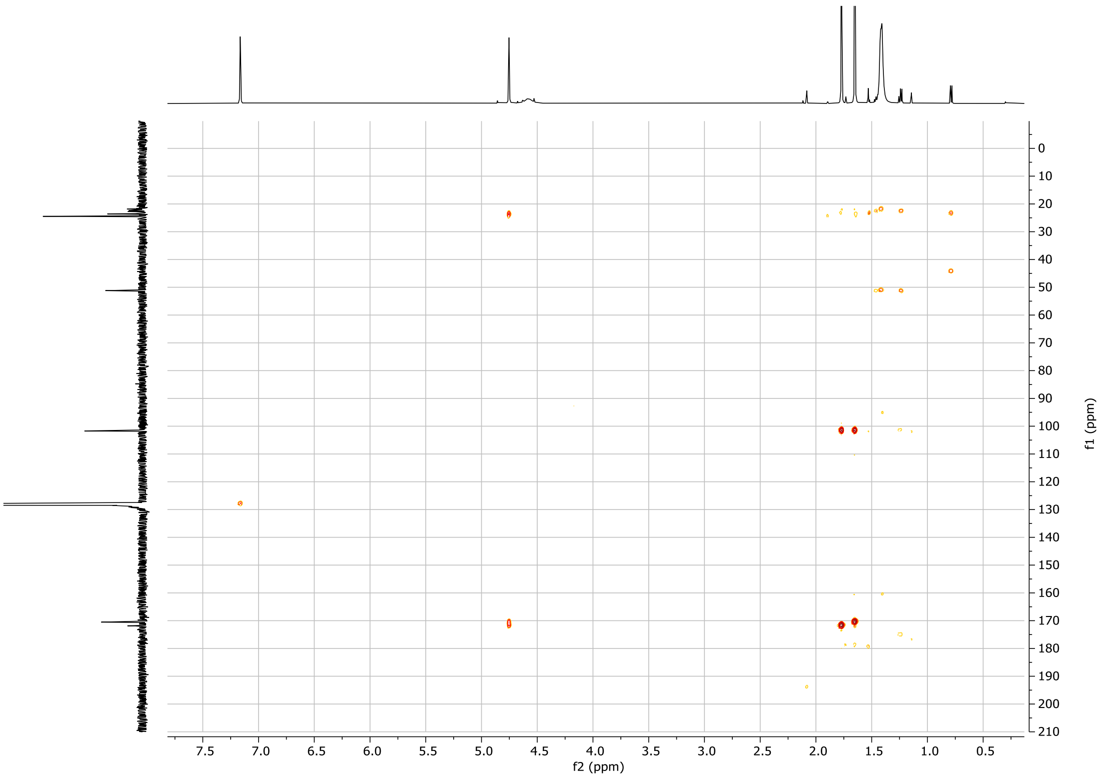

Figure S20: HMBC NMR spectrum of a C_6D_6 solution of (**[Al(^{i}Pr-acnac)$_2$Cl]**, (**3**).

[Al(MeCN(Ph)CHC=OMe)$_2$Cl] ([Al(Ph-acnac)$_2$Cl], **4**)

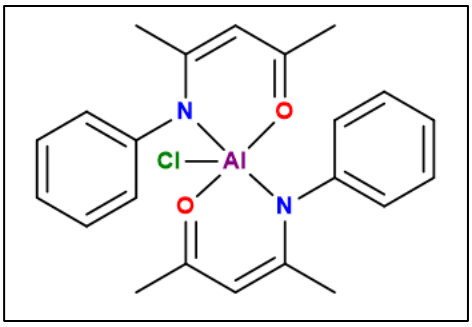

LiHMDS (1.00 g, 5.98 mmol) in toluene (20 mL) was added to a solution of Ph-acnacH (1.11 g, 6.37 mmol) in toluene (20 mL) at −78 °C. The reaction mixture was stirred at room temperature overnight to form a cloudy, pale-yellow solution. The volatiles were removed in vacuo to produce a pale-yellow solid, which was re-dissolved in toluene (20 mL) to reform a cloudy, pale-yellow solution which was added to a slurry of $AlCl_3$ (0.40 g, 3.00 mmol) in toluene (20 mL) at −78 °C. The reaction mixture was stirred at room temperature overnight to form a cloudy, pale-yellow solution, which was filtered, with the clear pale-yellow filtrate concentrated and stored at −20 °C. Overnight, white crystals suitable for single crystal X-ray measurement precipitated. The crystals were separated from the supernatant solution and dried under reduced pressure. Yield: 0.64 g (52%).

^{1}H NMR (C_6D_6, 500 MHz) δ 7.67, 7.16-6.99 (10H, m, Ar–H), 4.88 (3H, s, CH), 1.47 (6H, s, CH_3CO), 1.36 (6H, s, CH_3CN).

^{13}C{^{1}H} NMR (C_6D_6, 126 MHz) δ 176.87 (CH_3CO), 173.03 (CH_3CN), 149.01, 126.62, 125.23 (Ar–C), 100.74 (CH), 24.02 (CH_3CO), 23.41 (CH_3CN).

MS: m/z [ESI+] 411 $[M]^+$, 375 $[M – Cl]^+$.

CHN: Found (Calcd.) for $C_{22}H_{24}O_2N_2AlCl$: 62.83 (64.31), 5.80 (5.89), 6.54 (6.82).

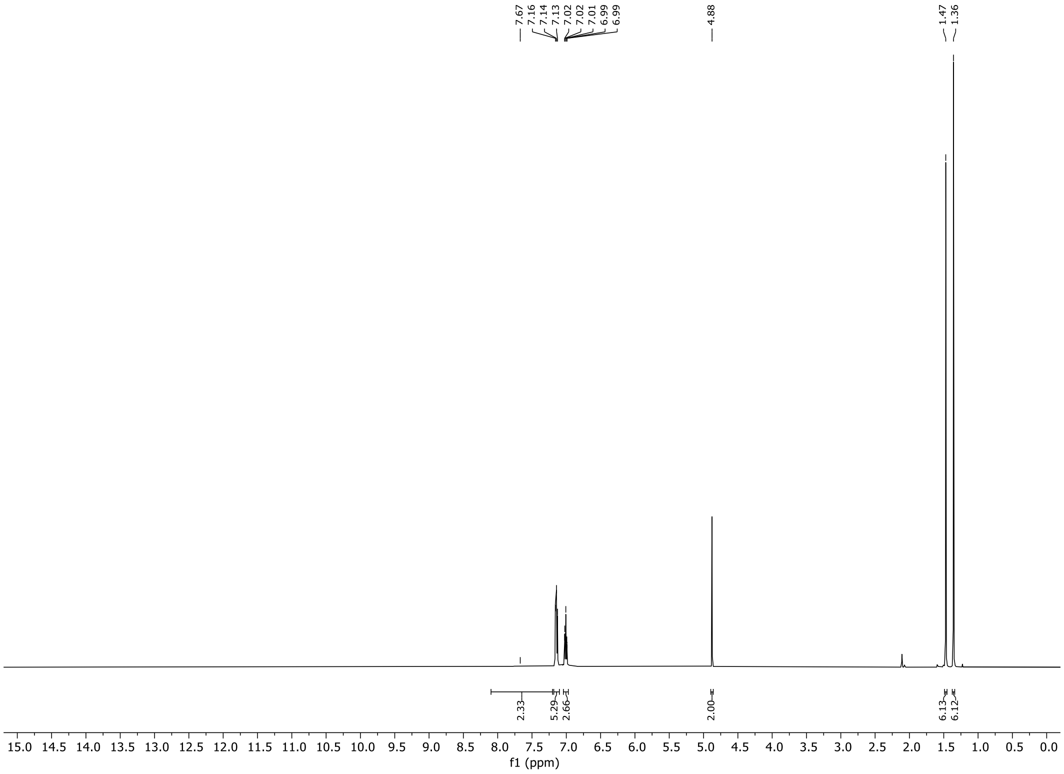

Figure S21: ^{1}H NMR (500 MHz) spectrum of a C_6D_6 solution of (**[Al(Ph-acnac)$_2$Cl]**, (**4**).

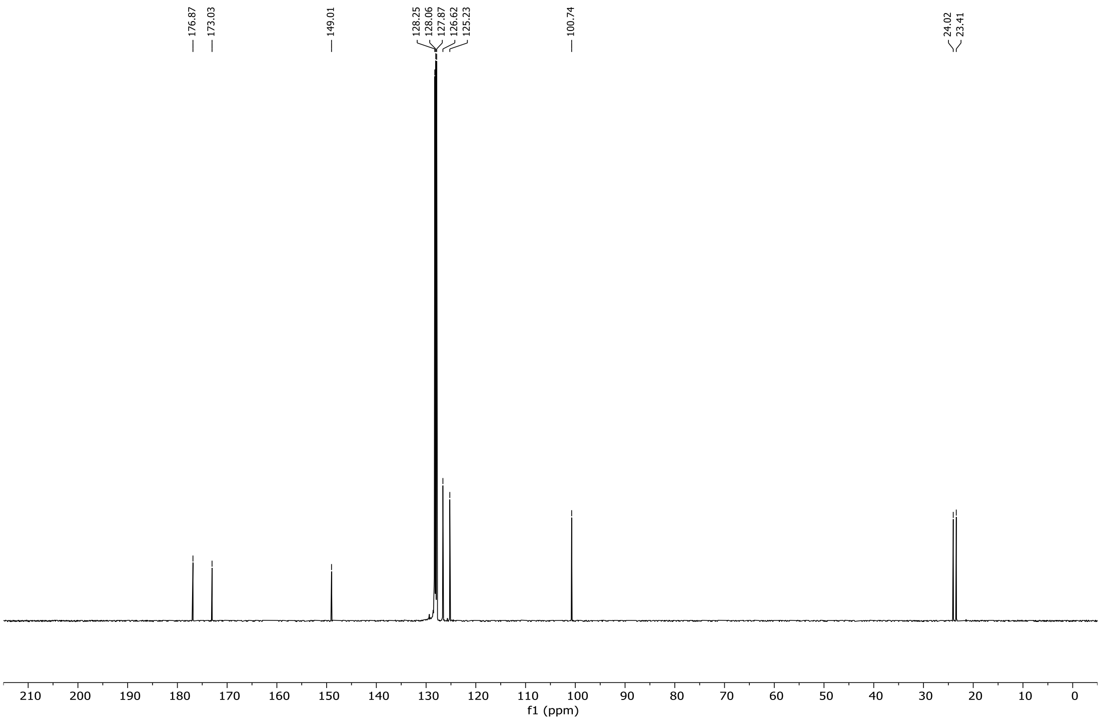

Figure S22: $^{13}C\{^{1}H\}$ NMR (126 MHz) spectrum of a C_6D_6 solution of (**[Al(Ph-acnac)$_2$Cl]**, (**4**).

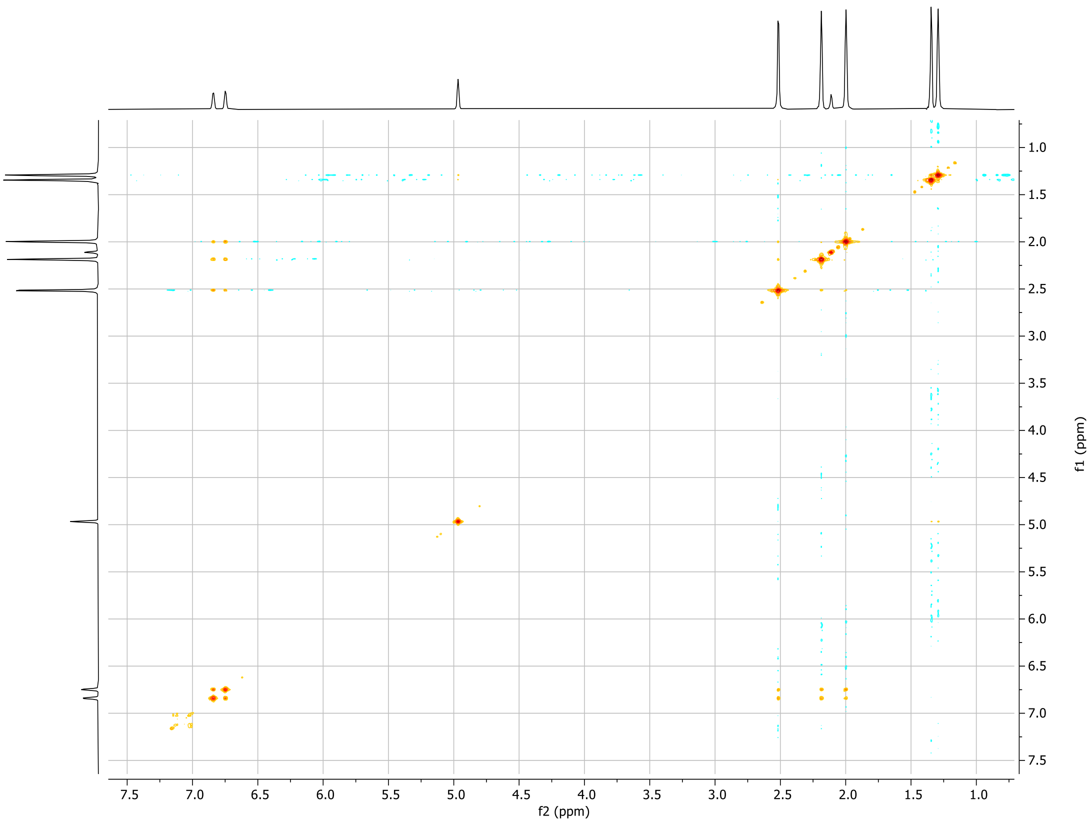

Figure S23: COSY NMR spectrum of a C_6D_6 solution of (**[Al(Ph-acnac)$_2$Cl]**, (**4**).

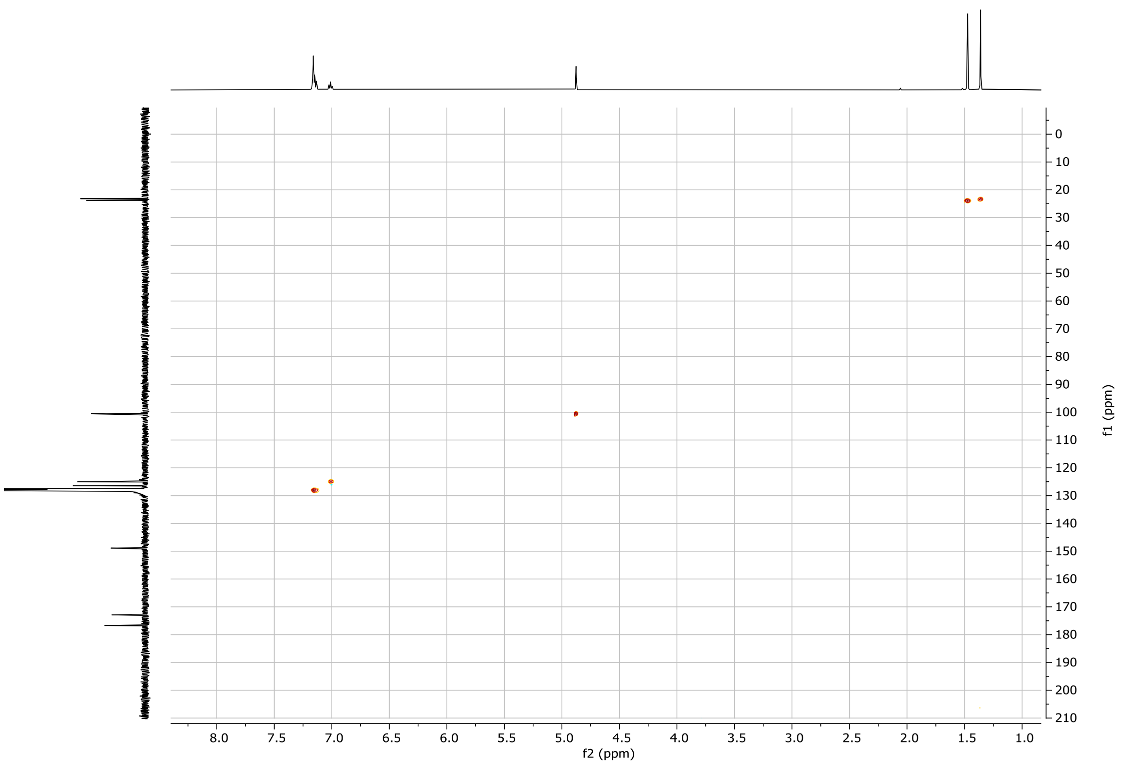

Figure S24: HSQC NMR spectrum of a C_6D_6 solution of (**[Al(Ph-acnac)$_2$Cl]**, (**4**).

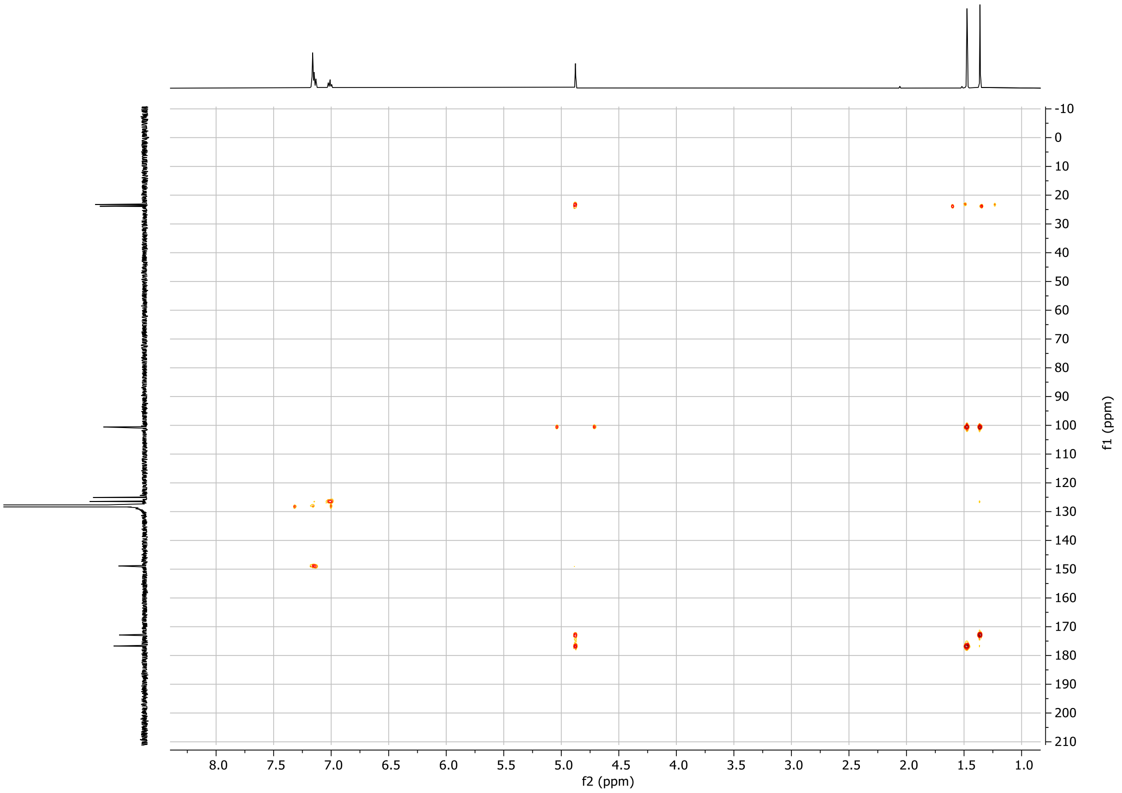

Figure S25: HMBC NMR spectrum of a C_6D_6 solution of (**[Al(Ph-acnac)$_2$Cl]**, (**4**).

[Al(MeCN(Mes)CHC=OMe)$_2$Cl] ([Al(Mes-acnac)$_2$Cl], **5**), where Mes = 2,4,6-trimethylphenyl

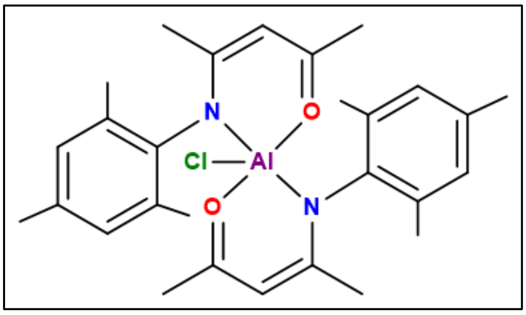

LiHMDS (1.00 g, 5.98 mmol) dissolved in toluene (20 mL)was added to a solution of Mes-acnacH (1.37 g, 6.33 mmol) in toluene (20 mL) at −78 °C. The reaction mixture was stirred at room temperature overnight to form a transparent, pale-yellow solution. The volatiles were removed in vacuo to produce a pale-yellow solid, which was re-dissolved in toluene (20 mL) and added to a slurry of $AlCl_3$ (0.40 g, 3.00 mmol) in toluene (20 mL) at −78 °C. The reaction mixture was stirred at room temperature overnight to form a cloudy, pale-yellow solution, which was filtered, with the clear pale-yellow filtrate concentrated and stored at −20 °C. Overnight, white crystals suitable for single crystal X-ray measurement precipitated. The crystals were separated from the supernatant solution and dried under reduced pressure. Yield: 0.78 g (53%).

^{1}H NMR (C_6D_6, 500 MHz) δ 6.84 (1H, s, Ar–H), 6,75 (1H, s, Ar–H), 4.97 (2H, s, CH), 2.52 (3H, s, CH_3), 2.19 (3H, s, CH_3), 2.00 (3H, s, CH_3), 1.35 (6H, s, CH_3CO), 1.29 (6H, s, CH_3CN).

^{13}C{^{1}H} NMR (C_6D_6, 126 MHz) δ 181.53 (CH_3CO), 176.50 (CH_3CN), 146.01, 134.13, 133.55, 131.23, 129.81, 128.53, 128.35 (Ar–C), 99.91 (CH), 24.24 (CH_3CO), 23.80 (CH_3CN), 20.86, 19.45, 18.96 (CH_3).

MS: m/z [ESI-] 466 $[M–Al–H]^-$.

CHN: Found (Calcd.) for $C_{28}H_{36}O_2N_2AlCl$: 66.47 (67.94), 7.27 (7.33), 5.50 (5.66).

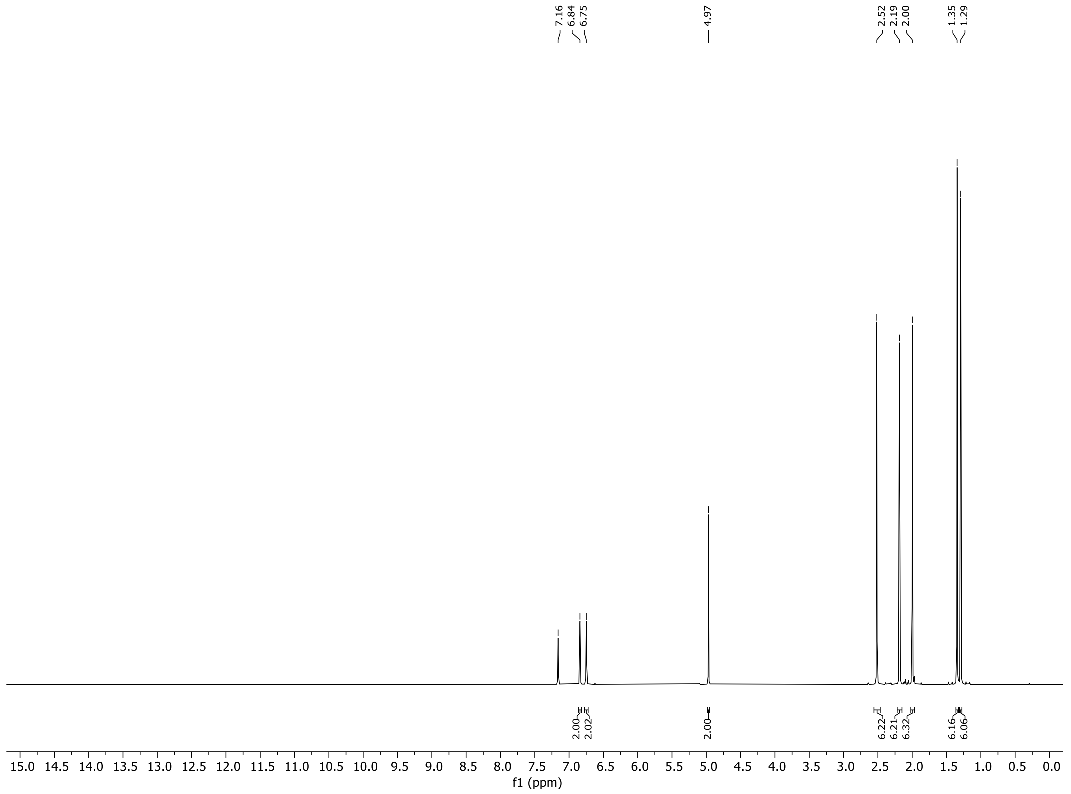

Figure S26: ^{1}H NMR (500 MHz) spectrum of a C_6D_6 solution of (**[Al(Mes-acnac)$_2$Cl]**, (**5**).

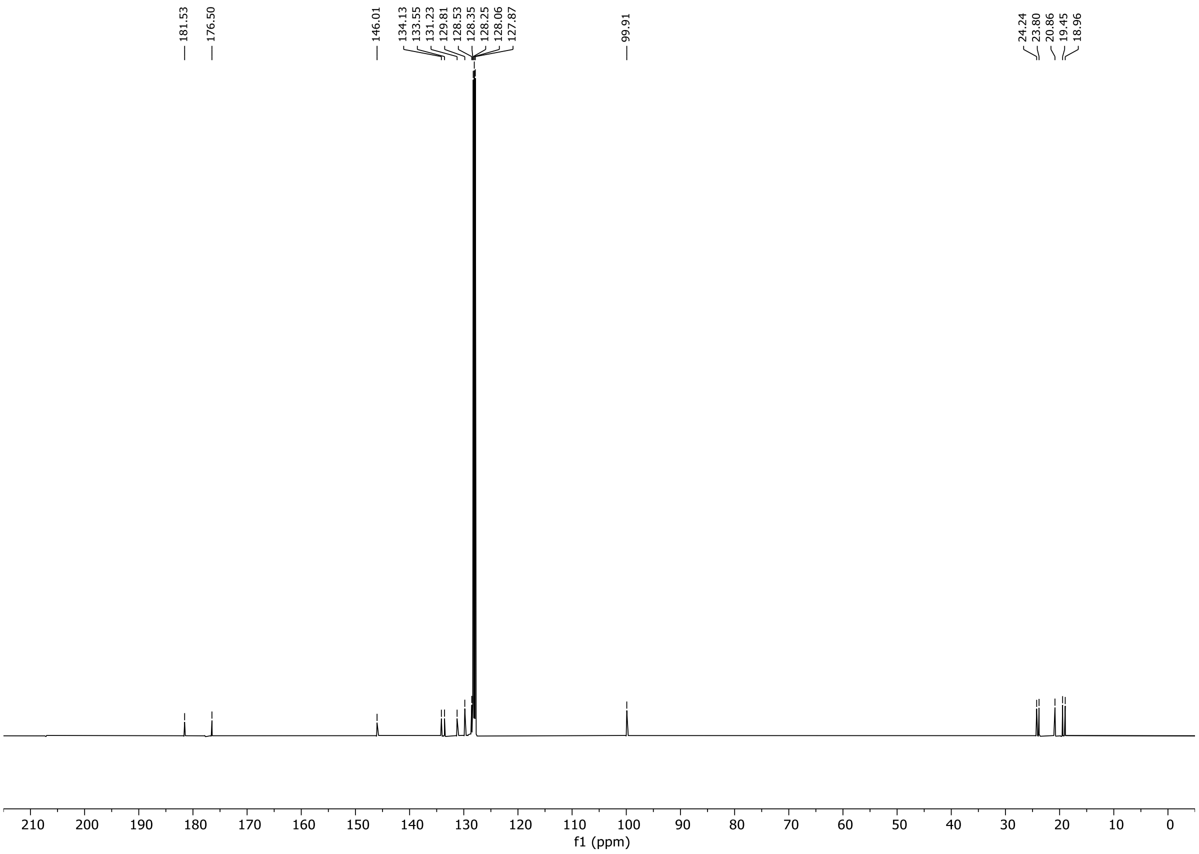

Figure S27: $^{13}C\{^{1}H\}$ NMR (126 MHz) spectrum of a C_6D_6 solution of (**[Al(Mes-acnac)$_2$Cl]**, (**5**).

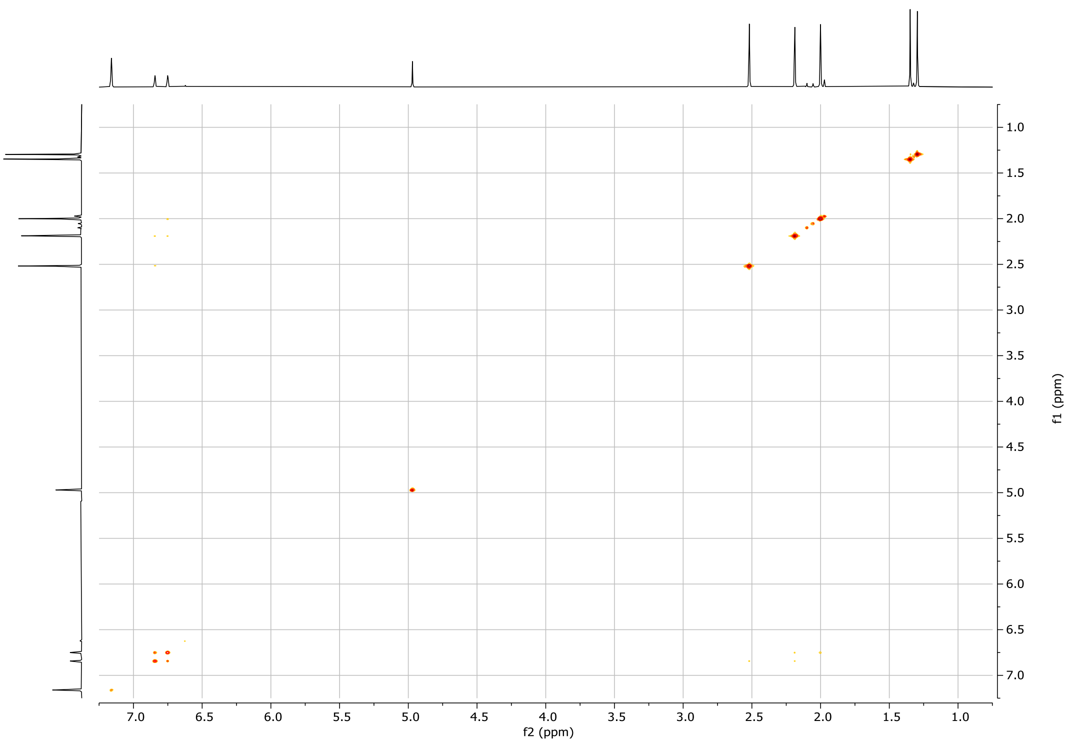

Figure S28: COSY NMR spectrum of a C_6D_6 solution of (**[Al(Mes-acnac)$_2$Cl]**, (**5**).

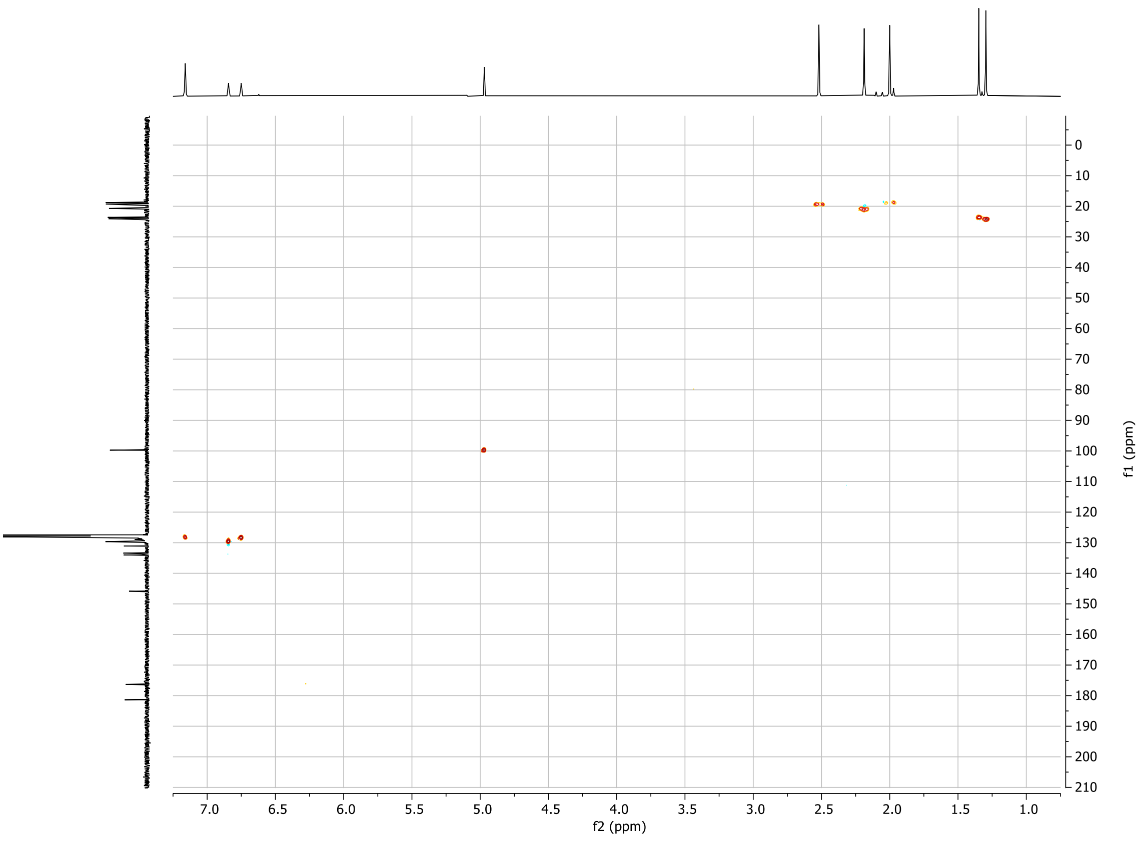

Figure S29: HSQC NMR spectrum of a C_6D_6 solution of (**[Al(Mes-acnac)$_2$Cl]**, (**5**).

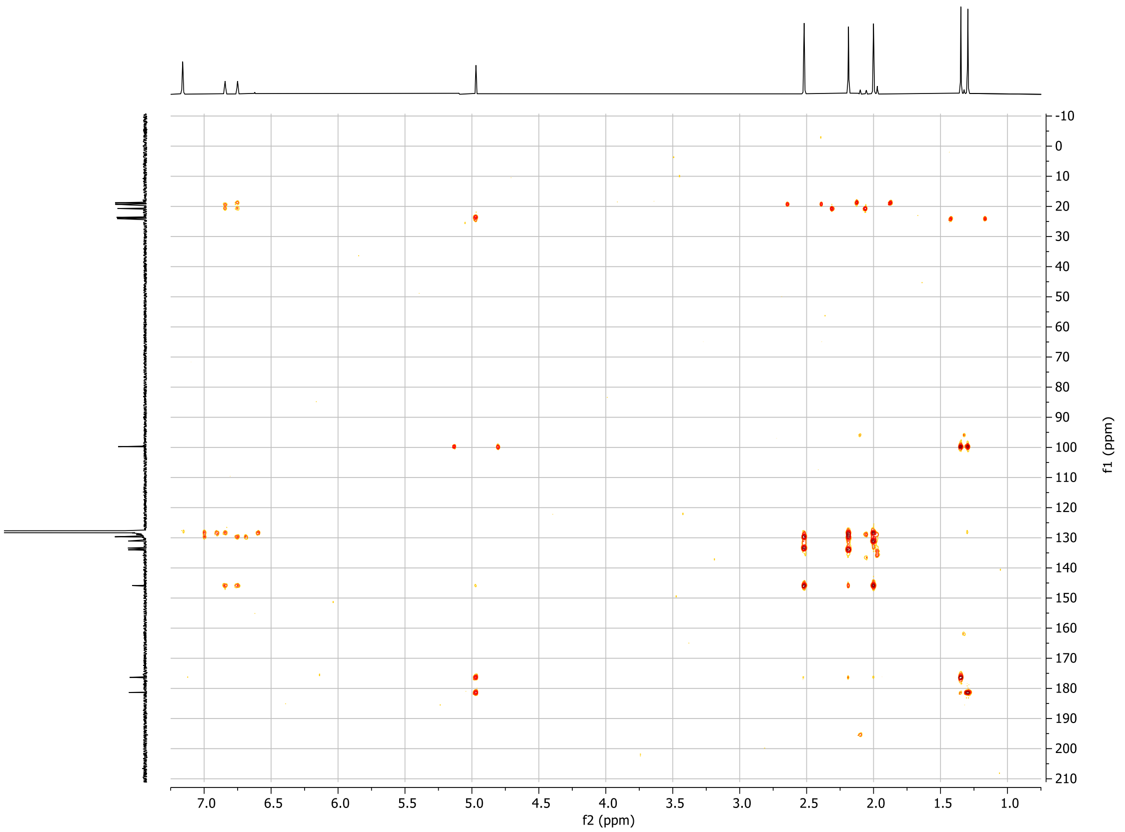

Figure S30: HMBC NMR spectrum of a C_6D_6 solution of (**[Al(Mes-acnac)$_2$Cl]**, (**5**).

Li[AlH$_2$(^{i}Pr-acnacH)AlH$_3$]$_n$ (**6**)

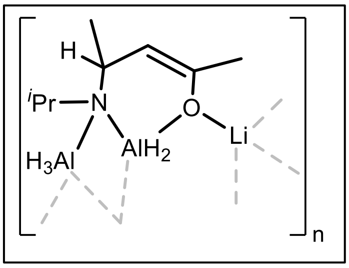

Diethyl ether (5 mL) was added of [Al(^{i}Pr-acnac)$_2$Cl] (0.153 mg, 0.447 mmol). In another Schlenk, diethyl ether (10 mL) was added to $LiAlH_4$ (50.9 mg, 1.34 mmol) to form a cloudy colourless solution. The $LiAlH_4$ was dropwise added to the [Al(^{i}Pr-acnac)$_2$Cl] mixture at −78 °C. The mixture was stirred at low temperature for one hour, then the cold bath was removed and the mixture stirred for further one hour. The colourless solution was filtered and the volatiles removed under reduced pressure to obtain a white solid. The solid was redissolved in ether and layered with hexane to obtain colourless crystals of the product. Crystalline yield: 25 mg (27%).

IR (cm^{-1}): 2969, 1834, 1738 [ν(Al−H)], 1658, 1373, 1314, 957, 887, 697, 613.

CHN: Found (Calcd.) for $C_8H_{18}Al_2LiNO$: 44.91(46.84), 9.25(8.84), 6.34(6.83).

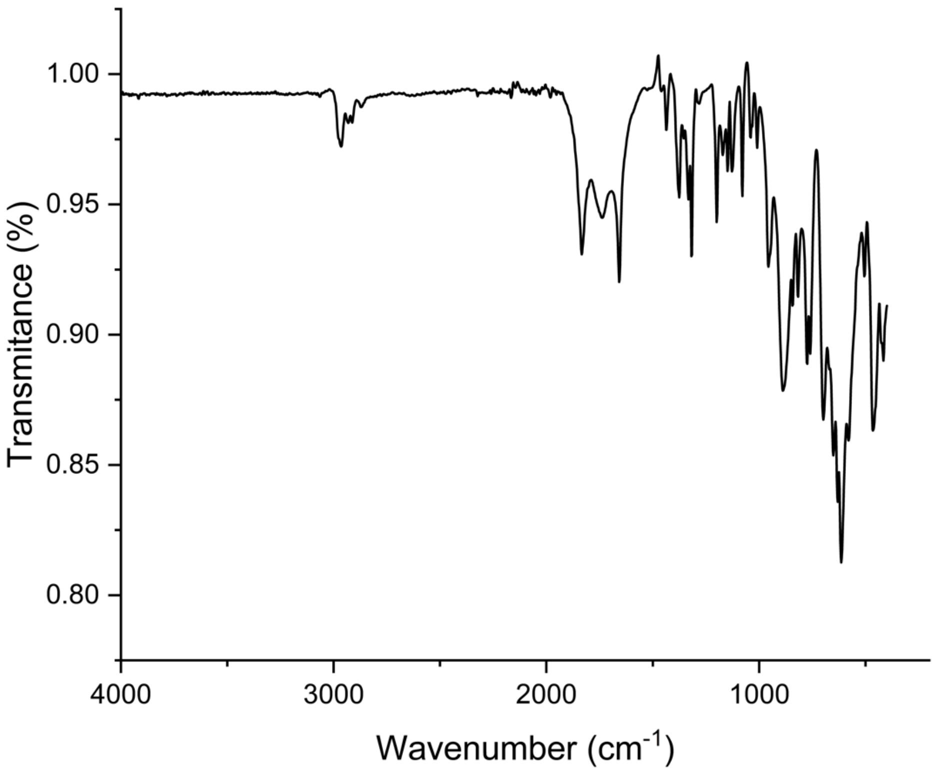

Figure S31: IR spectrum of **Li[AlH(^{i}Pr-acnacH)AlH$_3$]$_n$**, (**6**).

[AlH_2AlH_2(N-Mes)$_3$(AlH_2·Li(Et_2O)$_2$] (**8**)

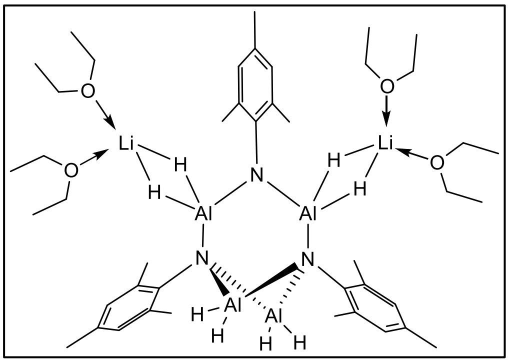

Diethyl ether (40 mL) was added to [Al(Mes-acnac)$_2$Cl] (0.725 g, 1.46 mmol) to form a cloudy solution. In another Schlenk, diethyl ether (50 mL) was added to $LiAlH_4$ (166.7 mg, 4.39 mmol). The cloudy solution of $LiAlH_4$ was dropwise added to [Al(Mes-acnac)$_2$Cl] at −78 °C. The mixture was stirred at low temperature for one hour, then the cold bath was removed and the mixture stirred for further 1.5 hours. The colourless solution was filtered and the volatiles removed under reduced pressure to form a foamy sticky solid that can be broken into a white powder when dipped into liquid nitrogen. The solid was redissolved in ether to obtain colourless crystals of the product at room temperature. Crystalline yield: 72.4 mg (6 %).

^{1}H NMR (C_6D_6, 500 MHz) δ 6.78 (1H, d, Ar–H), 6.74 (1H, d, Ar–H), 4.47 (s, AlH_2·Et_2O), 4.22 (br s, AlH_2–NMes), 3.18 (q, O(CH_2CH_3)$_2$), 2.81 (3H, s, *o*-CH_3), 2.77 (3H, s, *o*-CH_3), 2.10 (3H, s, *p*-CH_3), 1.04 (t, O(CH_2CH_3)$_2$).

^{13}C{^{1}H} NMR (C_6D_6, 126 MHz) δ 144.9 (C_{Ar}–N), 132.5, 132.07, 131.37, 131.27 (*o*-C_{Ar} and *m*-C_{Ar}), 130.89 (*p*-C_{Ar}), 66.38 (O(CH_2CH_3)$_2$), 25.79 (*o*-CH_3), 22.44 (*o*-CH_3), 20.64 (*p*-CH_3), 15.63 (O(CH_2CH_3)$_2$).

^{7}Li NMR (C_6D_6, 194 MHz) δ 0.44 (bs)

^{27}Al NMR (C_6D_6, 130 MHz) δ 70.92 (bs)

IR (cm^{-1}): 2975, 2906, 1783 [ν(Al–H)], 1654, 1662, 1472, 1198, 1136, 823, 738, 673.

CHN: Found (Calcd.) for $C_{43}H_{81}Al_4Li_2N_3O_4$: 59.92(62.53), 8.78(9.89), 5.24(5.09).

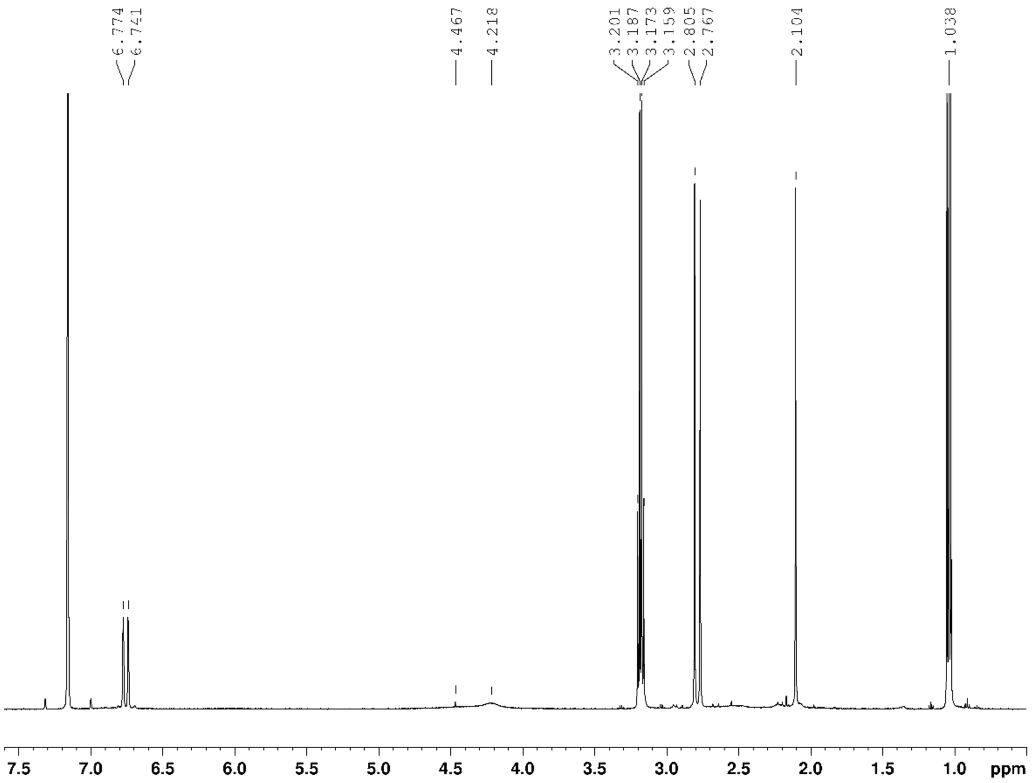

Figure S32: ^{1}H NMR (500 MHz) spectrum of a C_6D_6 solution of **[AlH$_2$AlH$_2$(N-Mes)$_3$(AlH$_2$·Li(Et$_2$O)$_2$)$_2$]** (**8**).

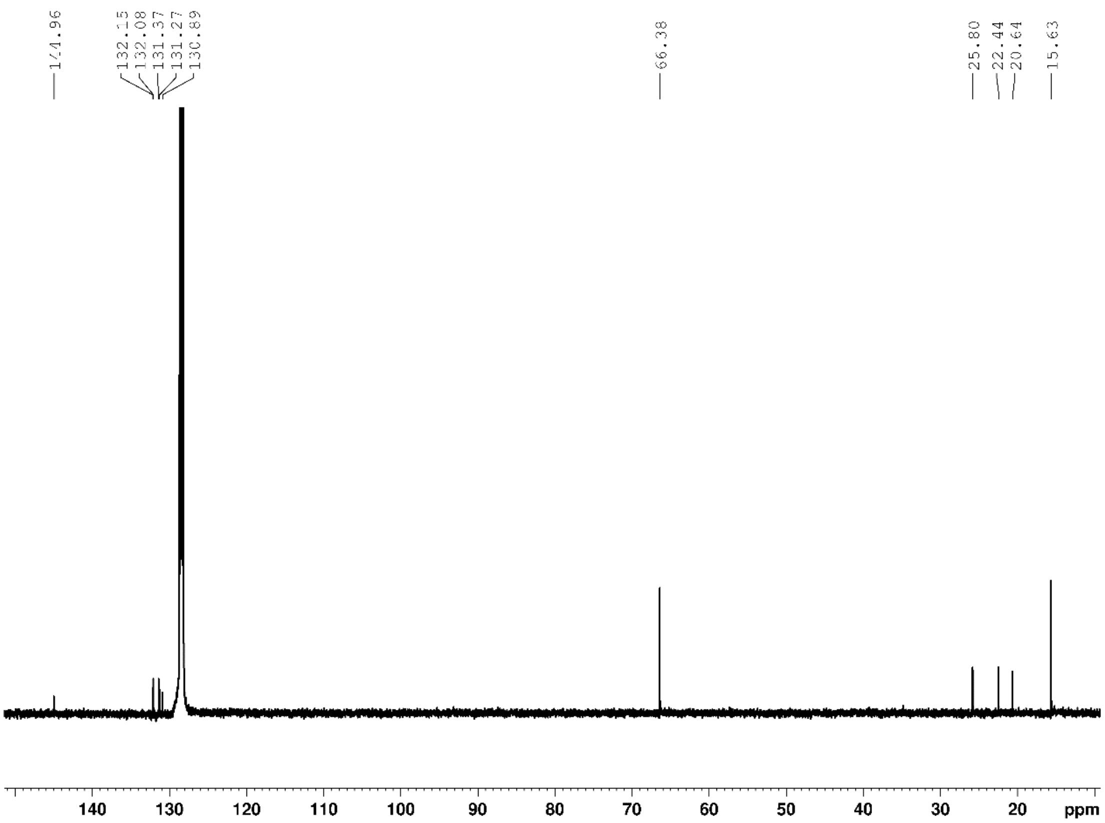

Figure S33: $^{13}C\{^{1}H\}$ NMR (126 MHz) spectrum of a C_6D_6 solution of **[AlH$_2$AlH$_2$(N-Mes)$_3$(AlH$_2$·Li(Et$_2$O)$_2$)$_2$]** (**8**).

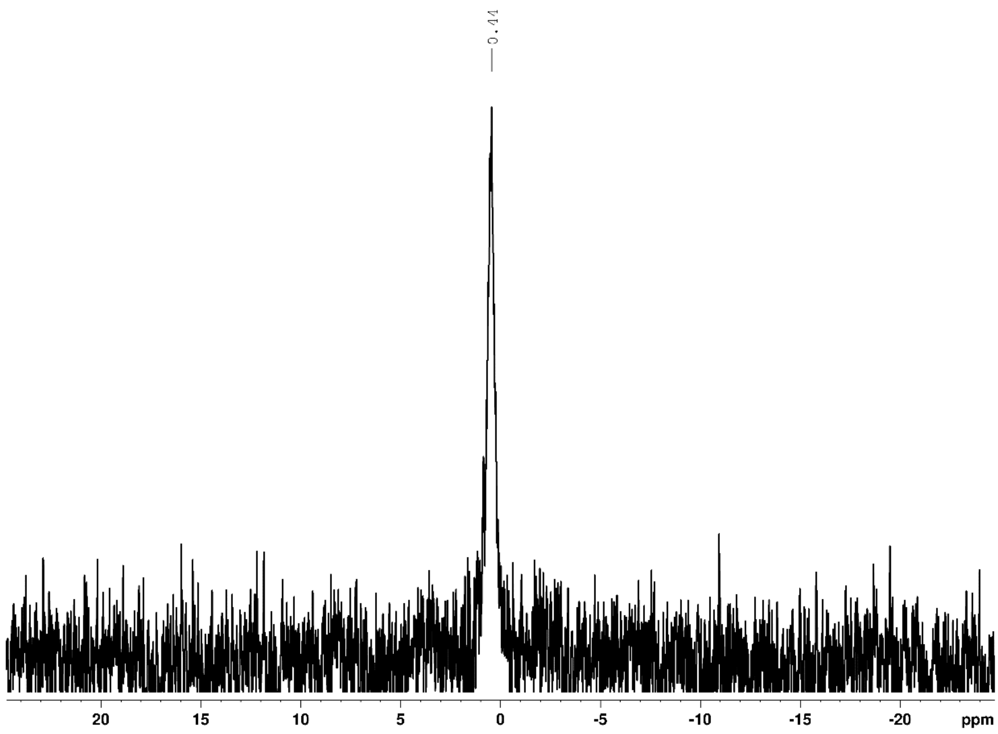

Figure S34: ^{7}Li NMR (194 MHz) spectrum of a C_6D_6 solution of **[AlH$_2$AlH$_2$(N-Mes)$_3$(AlH$_2$·Li(Et$_2$O)$_2$)$_2$]** (**8**).

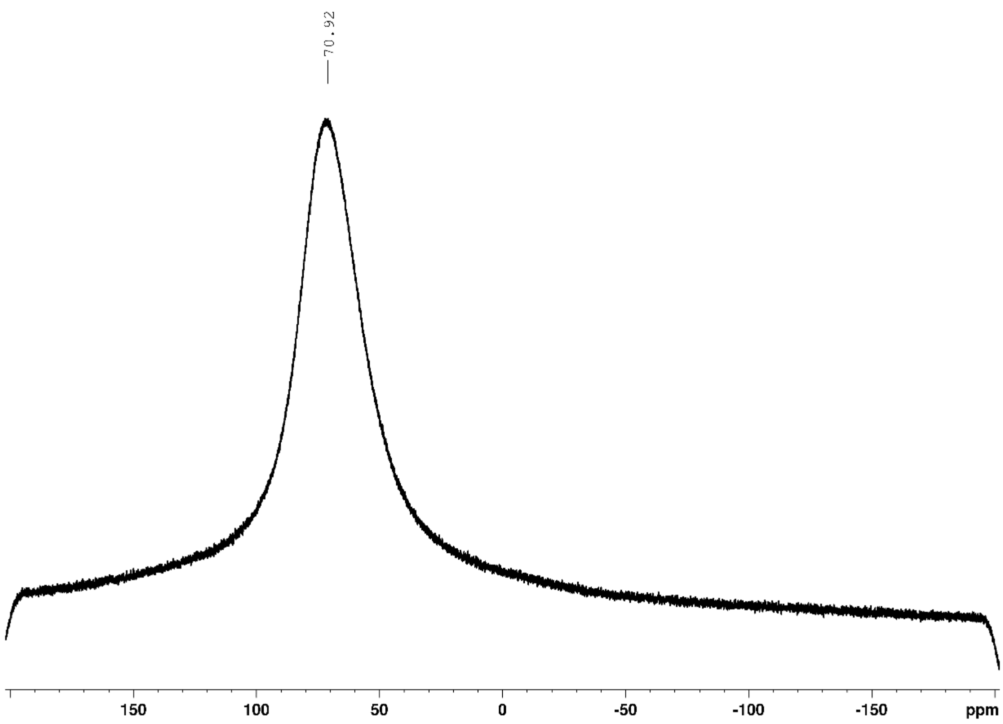

Figure S35: ^{27}Al NMR (130 MHz) spectrum of a C_6D_6 solution of **[AlH_2AlH_2(N-Mes)$_3$(AlH_2· Li(Et_2O)$_2$)$_2$]** (**8**).

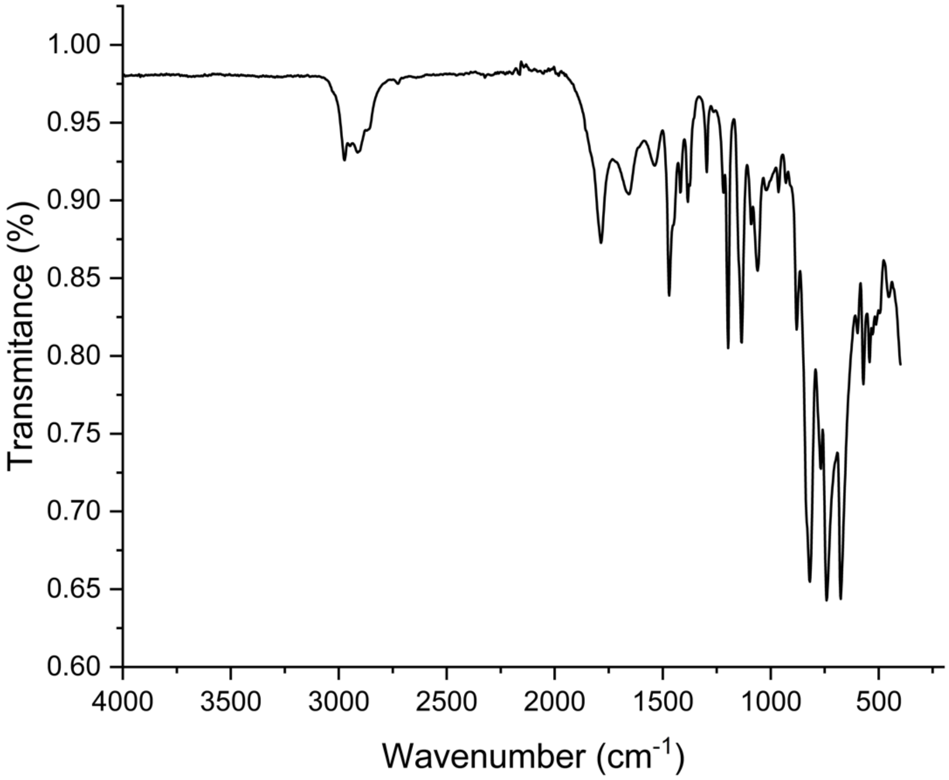

Figure S36: IR spectrum of **[AlH_2AlH_2(N-Mes)$_3$(AlH_2· Li(Et_2O)$_2$)$_2$]** (**8**).

Alternative route to obtain $[AlH_2AlH_2(N\text{-}Mes)_3(AlH_2\cdot Li(Et_2O)_2]$ (**8**)

2,4,6-trimethylaniline (NH_2–Mes) (0.5 mL, 3.70 mmol) was degassed prior use by the freeze, pump, thaw method. This was dissolved in diethyl ether (10 mL) in one Schlenk and added to another Schlenk containing $LiAlH_4$ (187 mg, 4.93 mmol) and diethyl ether (40 mL) at −78 °C. The mixture was stirred in the cold bath for 15 minutes, then the cold bath was removed and the mixture allowed to stir for further 40min. The resulting colourless solution was filtered the solvent removed to obtain a white foam that was turned into a solid by dipping the flask under vacuum in liquid nitrogen. Colourless crystals were obtained from diethyl ether at room temperature. Yield: 0.352 g (34%).

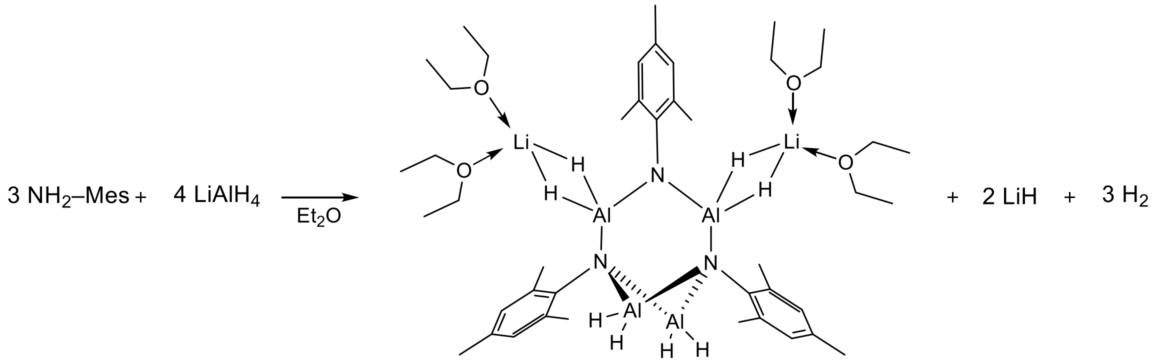

Figure S37: Reaction of three equivalents of NH_2–Mes with four equivalents of $LiAlH_4$ to alternatively obtain compound **8**.

2.3 Further scope: unsuccessful reactions of $LiAlH_4$ with aniline and various β-ketoiminates

2.3.1 Reaction of $LiAlH_4$ with aniline (NH_2–Ph):

Aniline (NH_2–Ph) (0.6 mL, 6.92 mmol) was degassed prior use by the freeze, pump thaw method and dissolved in 10 mL of diethyl ether in one Schlenk and added to another Schlenk containing $LiAlH_4$ (350 mg, 9.22 mmol) and diethyl ether (40 mL) at −78 °C. The mixture was stirred in the cold bath for 10 minutes, then the cold bath was removed and the mixture allowed to stir for further 40 min. The resulting colourless solution was filtered and the solvent removed to obtain a white foam that was turned into a solid by dipping the flask under vacuum in liquid nitrogen. 1.11g were obtained. ^{1}H NMR analysis (C_6D_6) of the products indicated the presence of intractable mixtures of the starting materials and byproducts.^{13}C{^{1}H} NMR revealed only signals for ether.

2.3.2 Reaction of $LiAlH_4$ with Me-acnacH (1:2)

Me-acnacH (4.34 g, 38.4 mmol) was dissolved in diethyl ether (20 mL) and added slowly to a stirred solution of $LiAlH_4$ (0.72 g, 19.2 mmol) in diethyl ether (20 mL) at −78 °C. The reaction mixture was allowed to gradually warm to room temperature and stirred overnight. A pale-

yellow solution formed, which was filtered to remove any undissolved material. The solvent was evaporated under reduced pressure, yielding a white solid. ^{1}H NMR analysis indicated the presence of intractable mixtures of starting materials and byproducts.

2.3.3 Reaction of $LiAlH_4$ with Et-acnacH (1:2)

Et-acnacH (4.88 g, 38.4 mmol) was dissolved in diethyl ether (20 mL) and added slowly to a cooled solution of $LiAlH_4$ (0.72 g, 19.2 mmol) in diethyl ether (20 mL) at −78 °C. The reaction mixture was allowed to gradually warm to room temperature and stirred overnight. A light yellow solution formed which was filtered to remove any undissolved material. The solvent was evaporated under reduced pressure, yielding a pale yellow solid. ^{1}H NMR analysis indicated the presence of intractable mixtures of starting materials and byproducts.

2.3.4 Reaction of $LiAlH_4$ with ^{i}Pr-acnacH (1:2)

iPr-acnacH (5.42 g, 38.4 mmol) was dissolved in diethyl ether (20 mL) and added dropwise to a cooled solution of $LiAlH_4$ (0.72 g, 19.2 mmol) in diethyl ether (20 mL) at −78 °C. The reaction mixture was allowed to gradually warm up to room temperature and warmed overnight, yielding a dark yellow solution. This was filtered to get rid of any undissolved materials and the solvent was evaporated under reduced pressure yielding a pale yellow solid. ^{1}H NMR analysis indicated the presence of intractable mixtures of starting materials and byproducts.

2.3.5 Reaction of $LiAlH_4$ with Ph-acnacH (1:2)

Ph-acnacH (6.72 g, 38.4 mmol) was dissolved in 20 mL of THF and added to a solution of $LiAlH_4$ (0.72 g, 19.2 mmol) in 20 mL of diethyl ether at −78 °C. The mixture was stirred overnight while warming to room temperature. The solution turned pale yellow, and the THF was removed under reduced pressure. The remaining solid was dissolved in diethyl ether, and the mixture was filtered. Crystallization from hexane was attempted, but no single crystals formed. The residue was a white solid that decomposed slowly when stored at room temperature. ^{1}H NMR revealed an intractable mixture of starting materials and products.

3. Thermal analysis

3.1. Thermal decomposition analysis of the bis(β-ketoiminate) aluminium chloride compounds [Al(R-acnac)$_2$Cl] (1-5)

The thermal decomposition properties of the bis(β-ketoiminate) aluminium chloride compounds **1**–**5** were investigated by thermogravimetric analysis (TGA) to access their suitability to act as precursors for the deposition of metallic features. Inspection of the thermogravimetric analysis of all [Al(R-acnac)$_2$Cl] complexes (Figure S36) reveal that a simple variation of the R group of the *N*-substituted ligand directly affects the thermal properties of the resulting complexes.

Promisingly, most complexes exhibit the beginnings of significant mass loss within the desired temperature window of 50–200 °C. Complex **1** (R = Me) exhibits significant mass loss beginning at 166 °C, complex **2** (R = Et) at 116 °C, **3** (R = ^{i}Pr) at 186 °C and **4** (R = pH) at 199 °C, while complex **5** (R = Mes), starts decomposing at 278 °C.

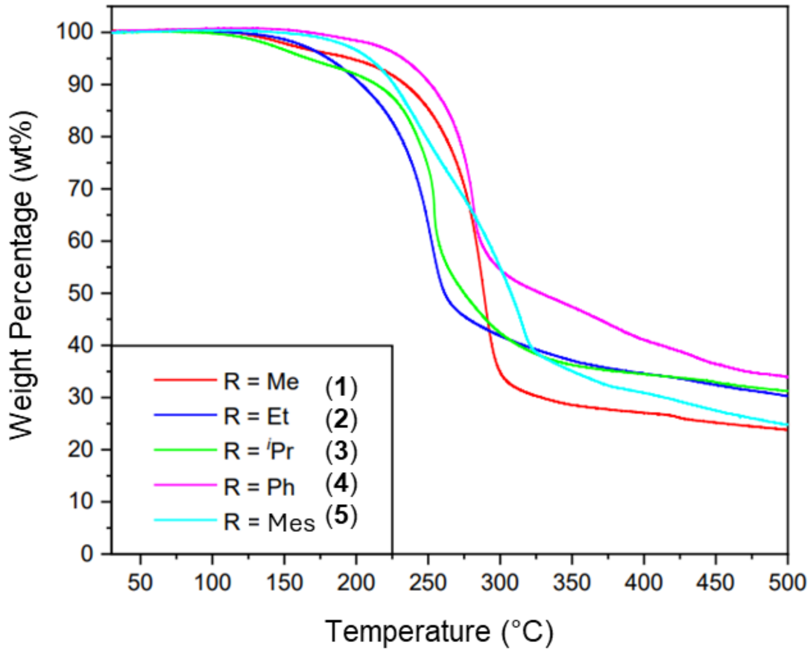

Figure S38: Overlapping thermograms of compounds **1**-**5**.

3.2. Thermal decomposition analysis of the aluminium hydride compounds 6

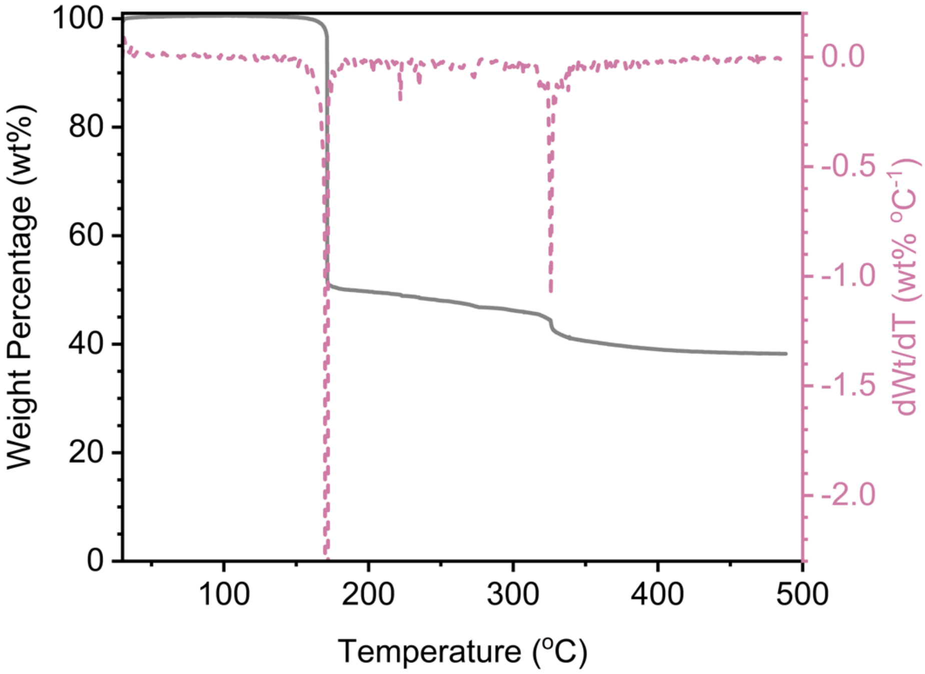

Figure S39: Overlapping thermogram (full grey line) and derivative (dashed pink line) of compound **6**.

4. X-Ray Diffraction

All PXRD experiments were performed on glass capillaries sealed with wax.

4.1. Room temperature PXRD

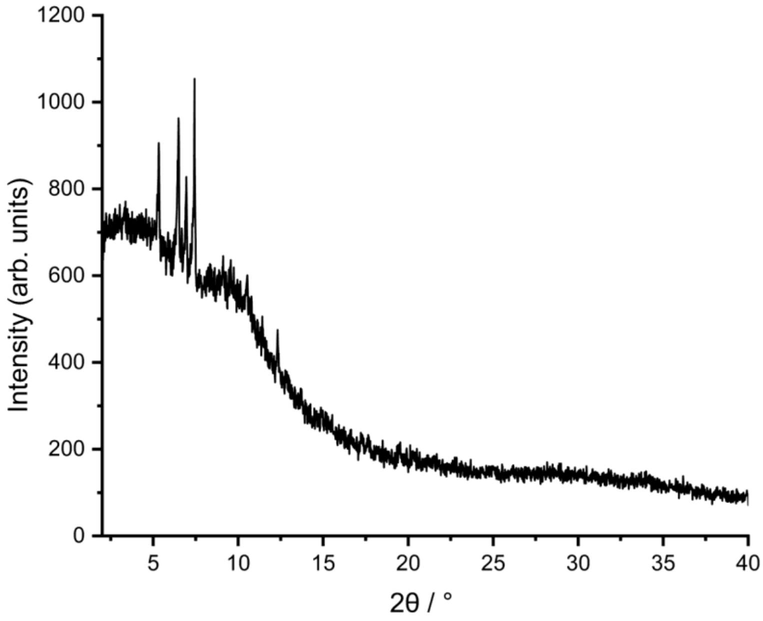

Figure S40: PXRD of **6** (measured with Mo kα).

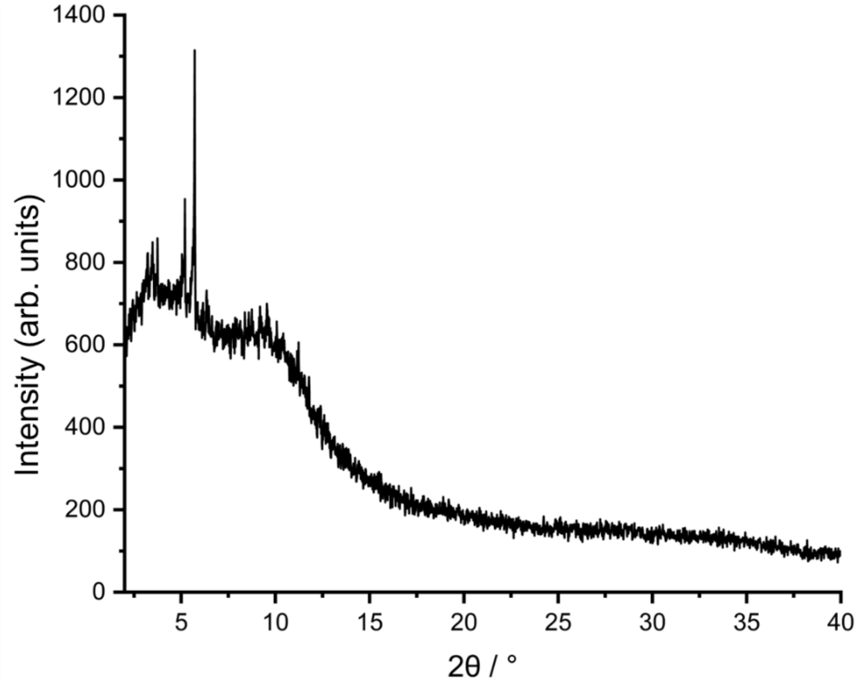

Figure S41: PXRD of crystalline **8** (measured with Mo kα).

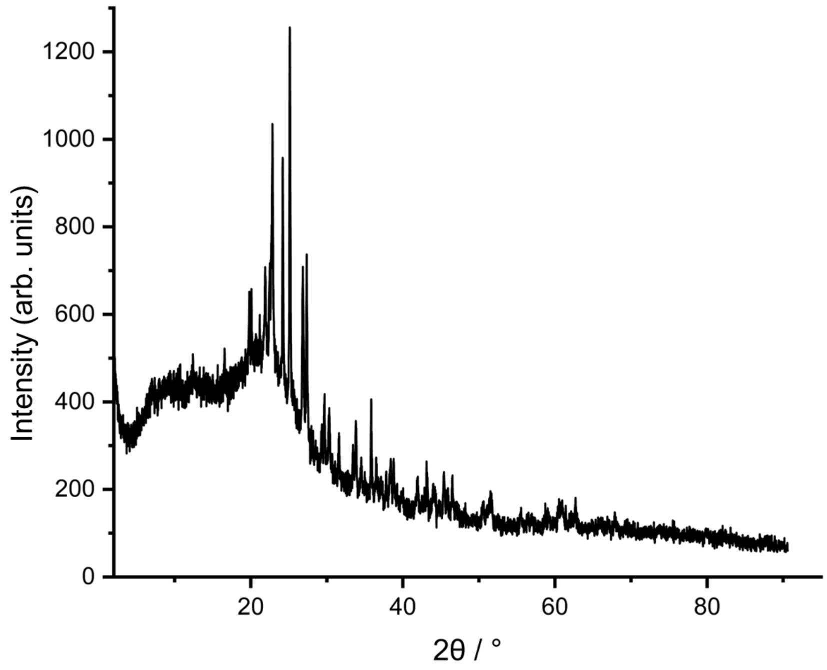

Figure S42: PXRD of the mixture of synthons of **8** (measured with Cu kα).

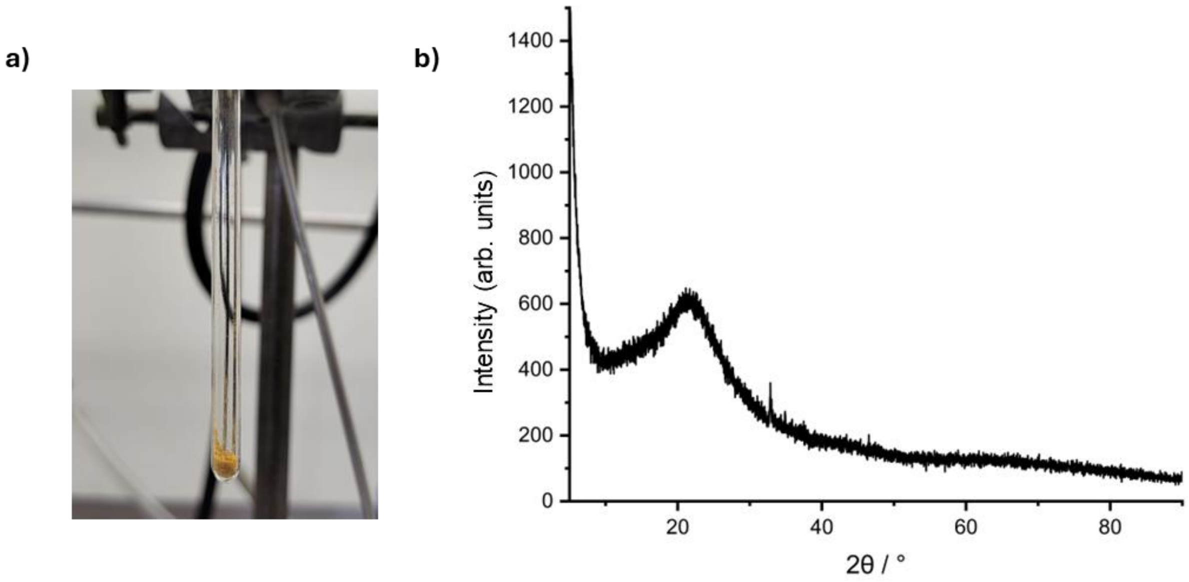

Figure S43: a) Cluster **8** after heating under nitrogen highlighting the clear colour change from white to light brown confirming the need to use vacuum to obtain a solid containing aluminium metallic; b) PXRD of the light brown solid (measured with Cu kα).

4.2. Variable-temperature PXRD

Variable-temperature PXRD (Mo kα) was measured for a wax-sealed sample under nitrogen. Changes onset at 50 °C, agreeing with the TGA behaviour, with the disappearance of the peak at 15.69° and emergence of a peak at 13.53°. The crystalline structure remains stable until 80 °C where several transformations are seen, firstly with the precursor peaks disappearing and a new peak emerging at 15.65°, while at 90 °C, the onset of weight loss in the TGA, a new crystalline phase is transiently formed with two peaks at 8.87° and 12.50°, which are lost at 100 °C, where only the 13.53° peak remains, followed at 110 °C by the formation of a peak at 15.50°. The structure then remains stable to 150 °C and ex-situ heating to 200 °C (measured with Cu kα) shows that this final phase remains stable.

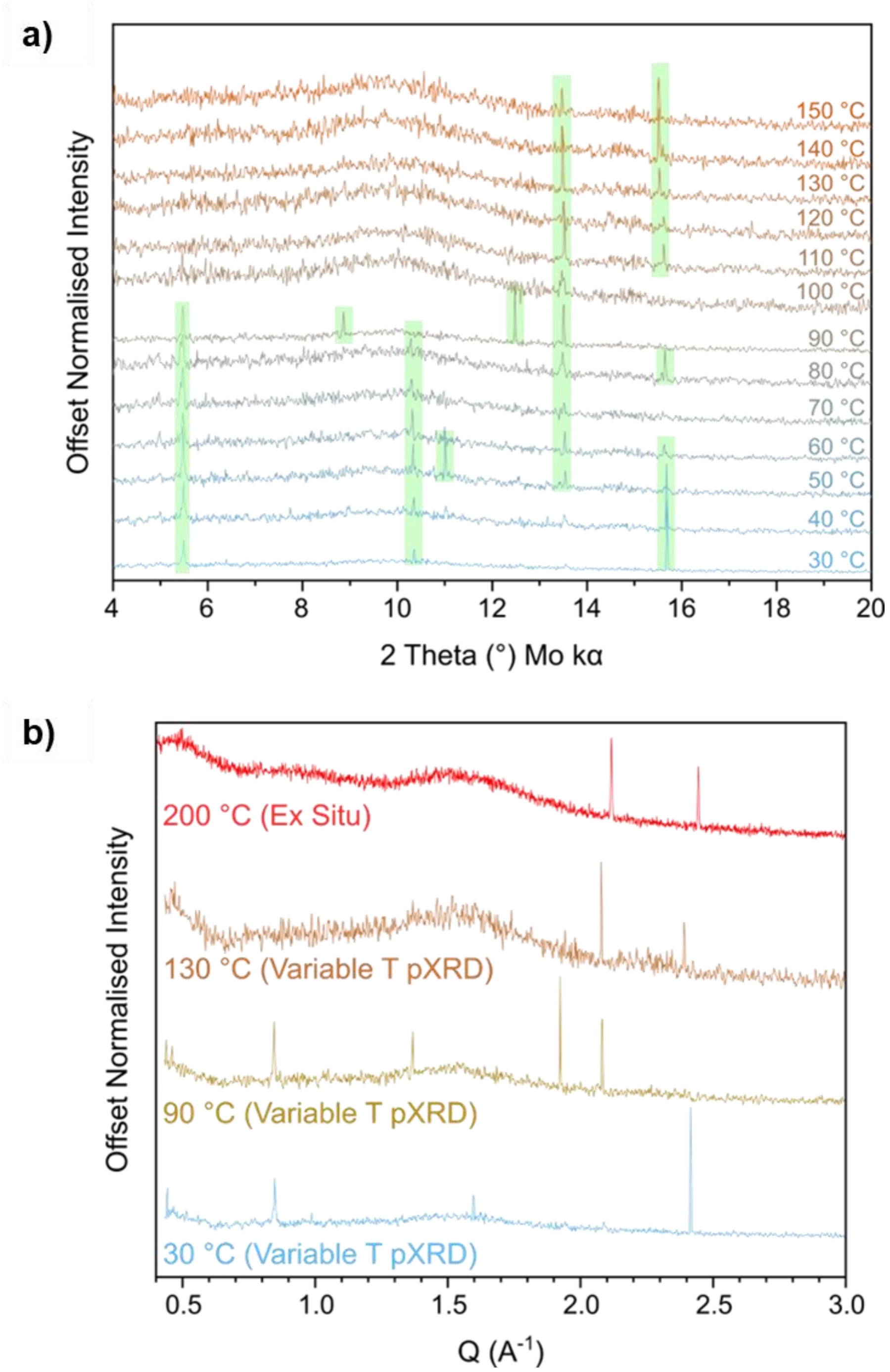

Figure S44: Degradation of **8** under nitrogen atmosphere. (a) Variable-temperature PXRD of wax sealed capillary sample heated in 10 °C increments between scans, data normalised and offset for clarity. Green shaded backgrounds used to highlight emerging/lost peaks as temperature increases. (b) Sample of **8** heated to 200 °C under nitrogen and cooled to room temperature (measured by Cu PXRD), in comparison to data from variable temperate experiment (Mo PXRD), plotted versus Q to provide a common x-scale.

4.3 Grazing incidence XRD

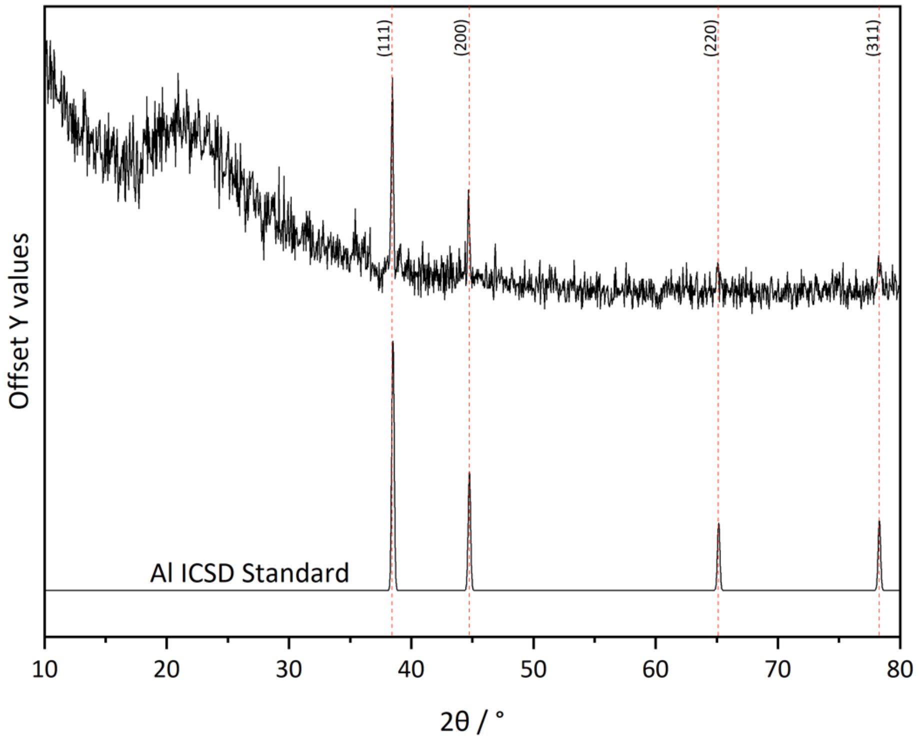

Figure S45: GI-XRD pattern of Aluminium film on glass, broad feature between 12-30 2 θ is the amorphous glass substrate.

5. X-Ray Photoelectron Spectroscopy (XPS)

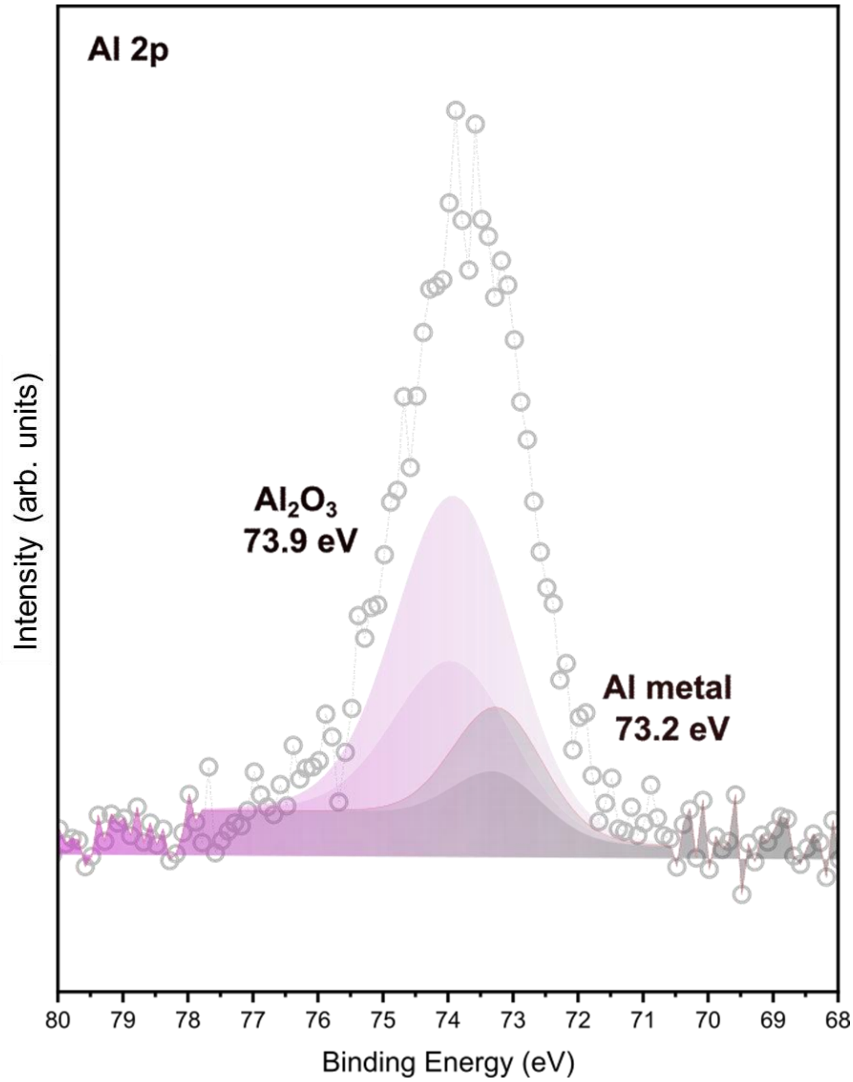

Figure S46: Al 2p XPS of the grey solid obtained from **8**: aluminium metal (Al) at 73.2 eV and aluminium oxide (Al_2O_3) at 73.9 eV.

6. Air- and moisture stability test of cluster 8

6.1. Cluster 8 compared with dimethyl amine alane (DMEAA)

To probe the superior user-friendly character of cluster **8** compared to alanes, dimethylamine alane (DMEAA) was chosen as a model alane compound since it is more stable than AlH_3 alone. Both compounds were exposed to air and dropped onto a tissue and their stability was evaluated.

When a hexane solution of DMEAA was used, signs of pyrophoricity could already be identified as soon as the port of the glove box was opened to air as the solution started fuming.

Subsequent dropping of the solution onto a tissue paper in air, lead to immediate ignition of the paper (Supplementary Movie 1). A comparable test with a toluene suspension of **8** leads to no visible effects (Supplementary Movie 2).

6.2. Cluster 8 exposed to air

When a C_6D_6 solution of cluster **8** is exposed to air, the formation of NH_2−Mes amine and other unidentified minor products is observed. Moreover, unlike when under nitrogen, the peaks corresponding to the ether molecules after exposure to air are found at 3.26 and 1.11 ppm, which are consistent with the uncoordinated species.

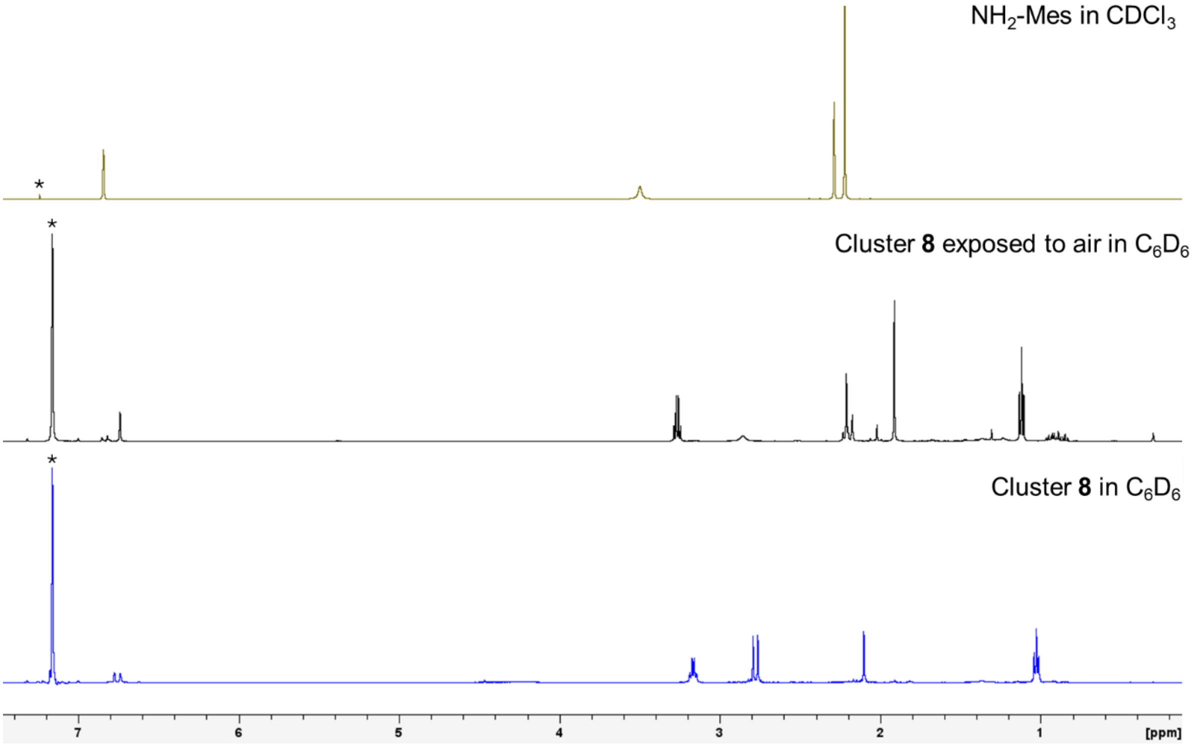

Figure S47: Stacked ^{1}H NMR spectra of cluster **8** in C_6D_6 (bottom), cluster **8** exposed to air in C_6D_6 (middle) and NH_2−Mes in C_6D_6 for comparison. NMR solvent residual peaks are marked with a star (*).

7. Transmission electron microscopy (TEM)

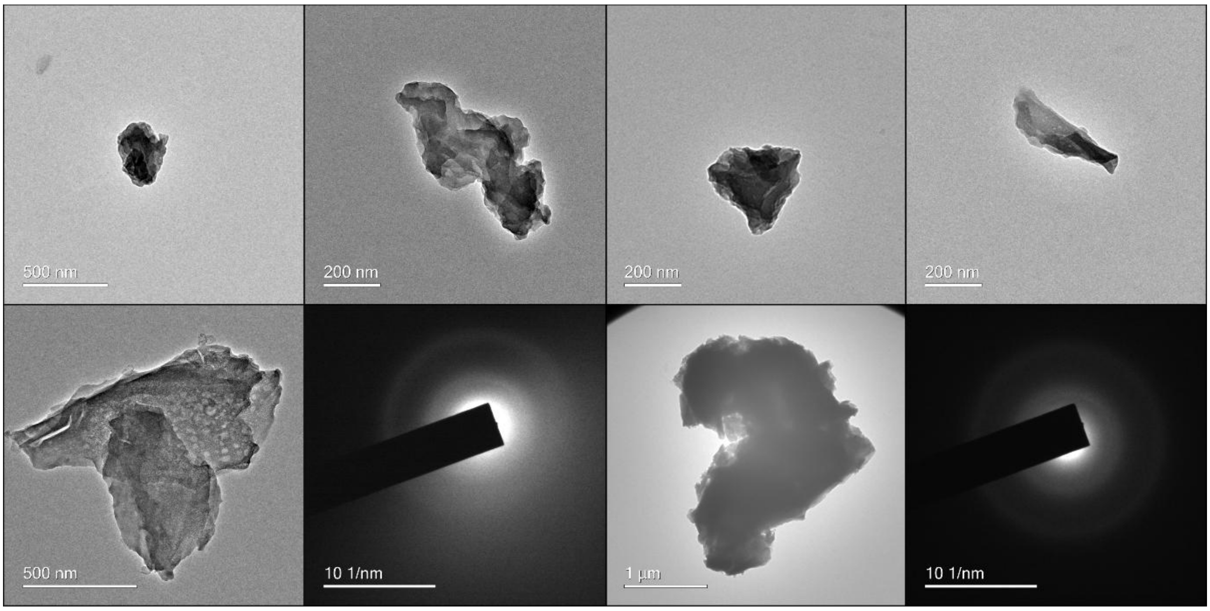

Figure S48: TEM micrographs of the cluster precursor **8**.

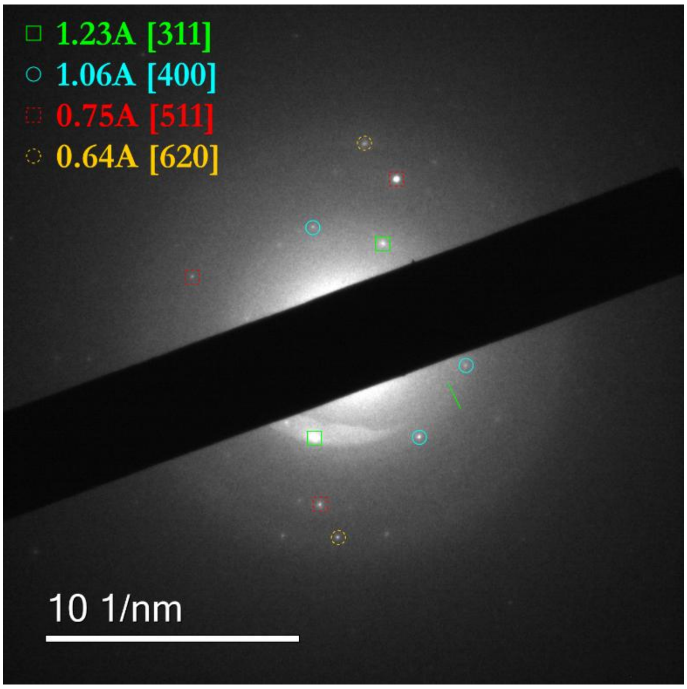

Figure S49: SAED assignment of the aluminium powder.

8. References

1. Pauling, L. The nature of the chemical bond. IV. The energy of single bonds and the relative electronegativity of atoms. *J Am Chem Soc* **54**, 3570–3582 (1932).

2. Yu, R. C., Hung, C. H., Huang, J. H., Lee, H. Y. & Chen, J. T. Four- and five-coordinate aluminum ketiminate complexes: Synthesis, characterization, and ring-opening polymerization. *Inorg Chem* **41**, 6450–6455 (2002).

3. Shukla, P., Gordon, J. C., Cowley, A. H. & Jones, J. N. Fluoroaryl-substituted ketiminate complexes of aluminum. *J Organomet Chem* **690**, 1366–1371 (2005).

4. Mears, K. L., Bhide, M. A., Knapp, C. E. & Carmalt, C. J. Investigations into the structure, reactivity, and AACVD of aluminium and gallium amidoenoate complexes. *Dalton Transactions* **51**, 156–167 (2021).

5. Pangborn, A. B., Giardello, M. A., Grubbs, R. H., Rosen, R. K. & Timmers, F. J. Safe and convenient procedure for solvent purification. *Organometallics* **15**, 1518–1520 (1996).

6. Douglas, S. P. *et al.* Tris(β-ketoiminate) Aluminium(III) Compounds as Aluminium Oxide Precursors. *Chempluschem* **88**, e202200411 (2023).

7. W. E. Garner, F. R. S. A. & Haycock, E. W. The thermal decomposition of lithium aluminium hydride. *Proc R Soc Lond A Math Phys Sci* **211**, 335–351 (1952).

8. Dolomanov, O. V., Bourhis, L. J., Gildea, R. J., Howard, J. A. K. & Puschmann, H. OLEX2: a complete structure solution, refinement and analysis program. *J. Appl. Cryst.* **42**, 339–341 (2009).

9. Bourhis, L. J., Dolomanov, O. V., Gildea, R. J., Howard, J. A. K. & Puschmann, H. The anatomy of a comprehensive constrained, restrained refinement program for the modern computing environment – Olex2 dissected. *Acta Cryst. A* **71**, 59–75 (2015).

10. Sheldrick, G. M. & IUCr. Crystal structure refinement with SHELXL. *Acta Cryst. C* **71**, 3–8 (2015).

11. Sheldrick, G. M. & IUCr. SHELXT – Integrated space-group and crystal-structure determination. *Acta Cryst. A* **71**, 3–8 (2015).
