# Supplementary material for: A non-pyrophoric precursor for the low temperature deposition of metallic aluminium
Source: Nat Commun. 2025 Jul 1;16:5645. doi: 10.1038/s41467-025-60786-2 (PMC12215716; doi:10.1038/s41467-025-60786-2)
Supplement: Supplementary file 2 — Description of Additional Supplementary Files [file 41467_2025_60786_MOESM2_ESM.pdf]

Description of Additional Supplementary Files

File name: Supplementary Movie 1
Description: Movie of DMEAA being exposed to air and burning.

File name: Supplementary Movie 2
Description: Movie of compound 8 being exposed to air and not combusting.
